# Soluble CD83 Alleviates Experimental Autoimmune Uveitis by Inhibiting Filamentous Actin-Dependent Calcium Release in Dendritic Cells

**DOI:** 10.3389/fimmu.2018.01567

**Published:** 2018-07-09

**Authors:** Wei Lin, Konrad Buscher, Beibei Wang, Zhichao Fan, Nannan Song, Peng Li, Yingying Yue, Bingqing Li, Cuiling Li, Hongsheng Bi

**Affiliations:** ^1^Institute of Basic Medicine, Shandong Academy of Medical Sciences, Jinan, China; ^2^Eye Institute of Shandong University of Traditional Chinese Medicine, Jinan, China; ^3^Department of Immunology, Shanghai Medical School, Fudan University, Shanghai, China; ^4^Department of Nephrology and Rheumatology, University Hospital Muenster, Münster, Germany; ^5^Division of Inflammation Biology, La Jolla Institute for Allergy and Immunology, La Jolla, United States

**Keywords:** soluble CD83, dendritic cells, calcium response, filamentous actin, T cells, experimental autoimmune uveitis

## Abstract

Soluble CD83 (sCD83) is the extracellular domain of the membrane-bound CD83 molecule, and known for its immunoregulatory functions. Whether and how sCD83 participates in the pathogenesis of uveitis, a serious inflammatory disease of the eye that can cause visual disability and blindness, is unknown. By flow cytometry and imaging studies, we show that sCD83 alleviates experimental autoimmune uveitis (EAU) through a novel mechanism. During onset and recovery of EAU, the level of sCD83 rises in the serum and aqueous humor, and CD83^+^ leukocytes infiltrate the inflamed eye. Systemic or topical application of sCD83 exerts a protective effect by decreasing inflammatory cytokine expression, reducing ocular and splenic leukocyte including CD4^+^ T cells and dendritic cells (DCs). Mechanistically, sCD83 induces tolerogenic DCs by decreasing the synaptic expression of co-stimulatory molecules and hampering the calcium response in DCs. These changes are caused by a disruption of the cytoskeletal rearrangements at the DC–T cell contact zone, leading to altered localization of calcium microdomains and suppressed T-cell activation. Thus, the ability of sCD83 to modulate DC-mediated inflammation in the eye could be harnessed to develop new immunosuppressive therapeutics for autoimmune uveitis.

## Introduction

Uveitis is an inflammatory eye disease that causes visual impairment and blindness (1). It encompasses different types of intraocular inflammation, and affects all parts of the eye including photoreceptors (2, 3). Conventional therapy relies on corticosteroids as the first-line therapy (3–5). However, long-term corticosteroid treatment can cause serious systemic and ocular side effects such as hypertension, diabetes, cataracts, and glaucoma. To prevent these complications, corticosteroid-sparing therapies with immunomodulatory activity have been developed, and clinical results are promising (4, 5).

A large number of inflammatory immune cells infiltrate into the eye, eventually causing a systemic immune disorder of uveitis (2, 6–8). Uveitogenic CD4^+^ T cells are crucial effectors that drive local tissue damage and the systemic progression (9–12). Type 1 helper T cells (Th1) and type 17 helper T cells (Th17) topically accumulate and secret IFN-γ and IL-17 to promote tissue damage (7, 8). Recently, mature dendritic cells (DCs) were reported to appear in the choroid during uveitic conditions, and mediate the inflammatory process of posterior uveitis (2, 6, 13). As unique antigen-presenting cells with the ability to activate naive T cells, DCs take center stage by inducing pathogenic T-cell activation (14, 15). Moreover, experimental autoimmune uveitis (EAU) can be induced by adoptively transferring mature DCs pulsed with uveitogenic antigens (16), and was ameliorated by injection of immature DCs through inhibiting uveitogenic CD4^+^ T-cell activation and differentiation (17, 18). Modulating DC biology could be a means to alleviate or prevent disease.

Soluble CD83 (sCD83) is the extracellular domain of the membrane-bound CD83 (mCD83) molecule, which has been described as a molecular marker for DC maturity (19–21). sCD83 is released by CD83^+^ cells, such as mature DCs, activated T cells and B cells, and activated NK cells (19, 20, 22, 23). Increasing evidence suggests that sCD83 could induce tolerogenic DCs with reduced expression of CD40, CD80, and CD83 (20, 24, 25). Also, it promotes the generation of indoleamine 2,3-dioxygenase (IDO) in DCs, leading to the induction of regulatory T cells (Tregs) (24). Thus, sCD83 might be an anti-inflammatory and immunosuppressive mediator (19, 24–28).

Calcium signaling as a proximal event during immune cells activation governs the function of DCs, including activation, maturation, migration, and the formation of immunological synapses with T cells (29–31). The modification of cytosolic-free calcium concentration can lead to immune suppression and impairment of DC activity (29, 30). Calcium release depends on the localization of calcium concentration microdomains during cell–cell contact (32, 33). ORAI1 is a key member of the calcium release-activated calcium channel protein family. Its localization at the immunological synapse is essential for calcium signaling (32, 33). Mitochondria in vicinity of calcium channels serve as calcium microdomains for calcium homeostasis in cells (34, 35). In this line, cytoskeletal filamentous actin (F-actin) plays a central role during cell–cell interaction, enabling the re-localization of calcium microdomains and adequate calcium release (33, 36–38). It is currently unknown whether sCD83 affects activity and calcium signaling in DCs.

Here, we show that sCD83 application reduces disease severity by hampering F-actin-depending calcium signaling in DCs and limiting DC-mediated CD4^+^ T cell activation. sCD83 might thus be a new potential therapeutic target in EAU.

## Materials and Methods

### Experimental Autoimmune Uveitis

Pathogen-free female C57BL/6 (B6) mice (6–8 week old, Peking Vital River Laboratory Animal Ltd., Beijing, China) maintained in specific pathogen-free conditions according to the guidelines of China National Institutes of Health. The experiments were approved by the ethics committee of Shandong Academy of Medical Sciences (Jinan, China).

The induction of EAU in C57BL/6 mice has been described previously (39–41). Briefly, C57BL/6 mice were subcutaneously immunized at six different locations (footpads, tail base, and flanks) with 350 µg of human interphotoreceptor retinoid-binding protein peptide_1–20_ (IRBP_1–20_, ChinaPeptides Co., Ltd., Suzhou, Jiangsu, China) that was emulsified in complete Freund’s adjuvant (Sigma-Aldrich Company, MA, USA). Concurrently, a single dose of 500 ng of pertussis toxin (PTX, Enzo Life Sciences, Farmingdale, YN, USA) was injected intraperitoneally. After immunization, the mice were examined every 3 days by histopathological examination and a Genesis-D retinal camera (Kowa Company Ltd., Japan) as previously reports (36, 37, 39). The disease was graded using a scoring system according as previously described (39, 42) (Table S1 in Supplementary Material). Mouse IL-10, IL-17, IFN-γ ELISA kits (eBioscience, San Diego, CA, USA) and sCD83 ELISA kits (USCN Life Science Inc., Wuhan, Hubei, China) were used for cytokine detection in serum or aqueous humor.

### sCD83 Treatment

On day 8 after immunization, sCD83 protein (10 μg/mouse), which consists of the extracellular domain of the mCD83 molecule fused with human IgG_1_ (Sino Biological Inc., Beijing, China), was either administered i.v. every other day or sCD83 (1 μg/mouse) was administered as eye drops every day. The eyes were harvested on 16 days for H&E histological staining and clinical examination. The spleen and the eyes were harvested and used for flow cytometry. The blood serum and aqueous humor were obtained to detect the concentration of cytokines.

### Optical Coherence Tomography (OCT) and Fundus Fluorescein Angiography (FFA)

After anesthesia, animals were injected i.p. with 200 µl of 2% fluorescein in PBS. FFA was performed using the autofluorescent channel of the Spectralis™ HRA (Heidelberg Engineering, Heidelberg, Germany). Spectralis optical coherence tomography (OCT) (Heidelberg Engineering, Heidelberg, Germany) was performed with five-line raster scans. A custom scan was centered on optic nerve with a rotation of 30° and a length of 6 mm. The Heidelberg eye explorer (version 1.7.1.0) was used with the viewing module (version 5.7.0.9) and the acquisition module (version 5.6.3.0). Images were exported and processed in Adobe Photoshop CS5.5 (Adobe Systems Inc., San Jose, CA, USA).

### Cells Isolation

The eyes were collected as previously reported (40, 43). Briefly, the lens and cornea of eyes were removed, and digested by collagenase (1 mg/ml) and DNAse (100 µg/ml) in RPMI-1640 (40). Splenocytes were obtained and Red Blood Cell Lysis Buffer (Solarbio Science & Technology Co., Ltd., Beijing, China) were used to obtain the Single cell suspension as previously reported (40, 43). Primary CD4^+^ T cells and DCs were obtained from the spleen and selected using a CD4-negative and CD11c-positive selection kit (Miltenyi Biotec, Bergisch Gladbach, Germany), respectively.

### Flow Cytometry

Aliquots of 1 × 10^6^ cells were stained with different monoclonal antibodies according to standard protocols. The cells were analyzed on a FACSVerse cytometer (BD Biosciences, San Diego, CA, USA). To assess intracellular cytokine expression, the prepared cells were stimulated by the leukocyte activation cocktail containing phorbol 12-myristate 13-acetate (PMA) and ionomycin (BD Biosciences, San Diego, CA, USA). Fluorescent antibodies of CD45 (clone 30-F-11), CD3ε (clone 145-2C11), CD8 (clone 53.6.7), CD4 (clone GK1.5), CD83 (clone Michel-19), CD80 (clone 16-10A), CD86 (clone GL1), CD54 (clone 3E2), CD11b (clone M1/70), ly6c (clone HK1.4), F4/80 (clone BM8), B220 (clone RA3-6B2), NK1.1 (clone PK136), MHC-II (clone M5/114.15.2), CD11c (clone N418), IFN-γ (clone XMG1.2), IDO (clone 2E2/IDO1), IL-10 (clone JES5-16E3), and IL-17 (clone ebio17B7) conjugated with the corresponding fluorescent dyes (eBioscience, San Diego, CA, USA) were used in the experiments.

### Confocal Imaging

Wild-type DCs or DC2.4 cell line (a kind gift from professor Yiwei Chu, Fudan University, Shanghai, China) were incubated with IRBP_1–20_ (10 ng/ml) and PTX (10 ng/ml) overnight. CD4^+^ T cells were purified from the spleen of IRBP_1–20_-immunized B6 mice and were stimulated with IRBP_1–20_ (10 µg/ml) in the presence of 1 × 10^7^ irradiated syngeneic spleen cells as APCs in the presence of IL-2 (10 ng/ml), and then antigen-specific T cells were obtained by magnetic beads. CD4^+^ T cells were co-cultured with antigen-pulsed mature DCs (T cell:DC ratio = 10:1) for 30 min, until the T-cell–DC contact had been established. Then, the cells were fixed in PBS/4% paraformaldehyde for 10 min, followed by incubation with PBS/0.1 M of glycine for 3 min and blocking with PBS/2% bovine serum albumin buffer for 20 min. Next, the cells were stained with a 1:100 dilution of anti-ORAI1 antibody (Abcam, Cambridge, MA, USA) for 60 min. After washing, the cells were analyzed using confocal microscopy. For F-actin staining or mitochondrial labeling, TRITC Phalloidin (Molecular Probes, Carlsbad, CA, USA) or MitoTracker^®^ Green^FM^ (Molecular Probes, Carlsbad, CA, USA) was used as previously reported (33, 36). The pCAG-LifeAct-RFP plasmid was used for F-actin visualization in DCs according to the manufacturer’s instructions (ibidi, Martinsried, Germany).

Calcium imaging of live cells was performed using Fluo-3 (Molecular Probes, Carlsbad, CA, USA) as previously described and measured by an equation previously reported (36, 44). Time-lapse scanning was performed with a 40 s acquisition interval, and sustained for 10 min.

Images of the cells were taken with a confocal microscope (LMS 780, Zeiss, Germany) equipped with an APO oil immersion objective lens (63×, NA = 1.40) (36, 44). The images were analyzed with the Imaris software (Bitplane AG, Zurich, Switzerland) and Image J (National Institutes of Health, Bethesda, MD, USA).

### Statistical Analysis

Data analysis was performed using GraphPad Prism 5 (GraphPad Software, San Diego, CA, USA). Two-tailed Student’s *t*-test or one-way ANOVA was used as parametric tests. Mann–Whitney *U* tests or Kruskal–Wallis test were used as nonparametric tests. Data were represented as mean ± SEM *p*-values <0.05 (*), 0.01 (**), and 0.001 (***) were considered to be significant.

## Results

### Increase of CD83^+^ Leukocytes and sCD83 During EAU

**Figure 1:**
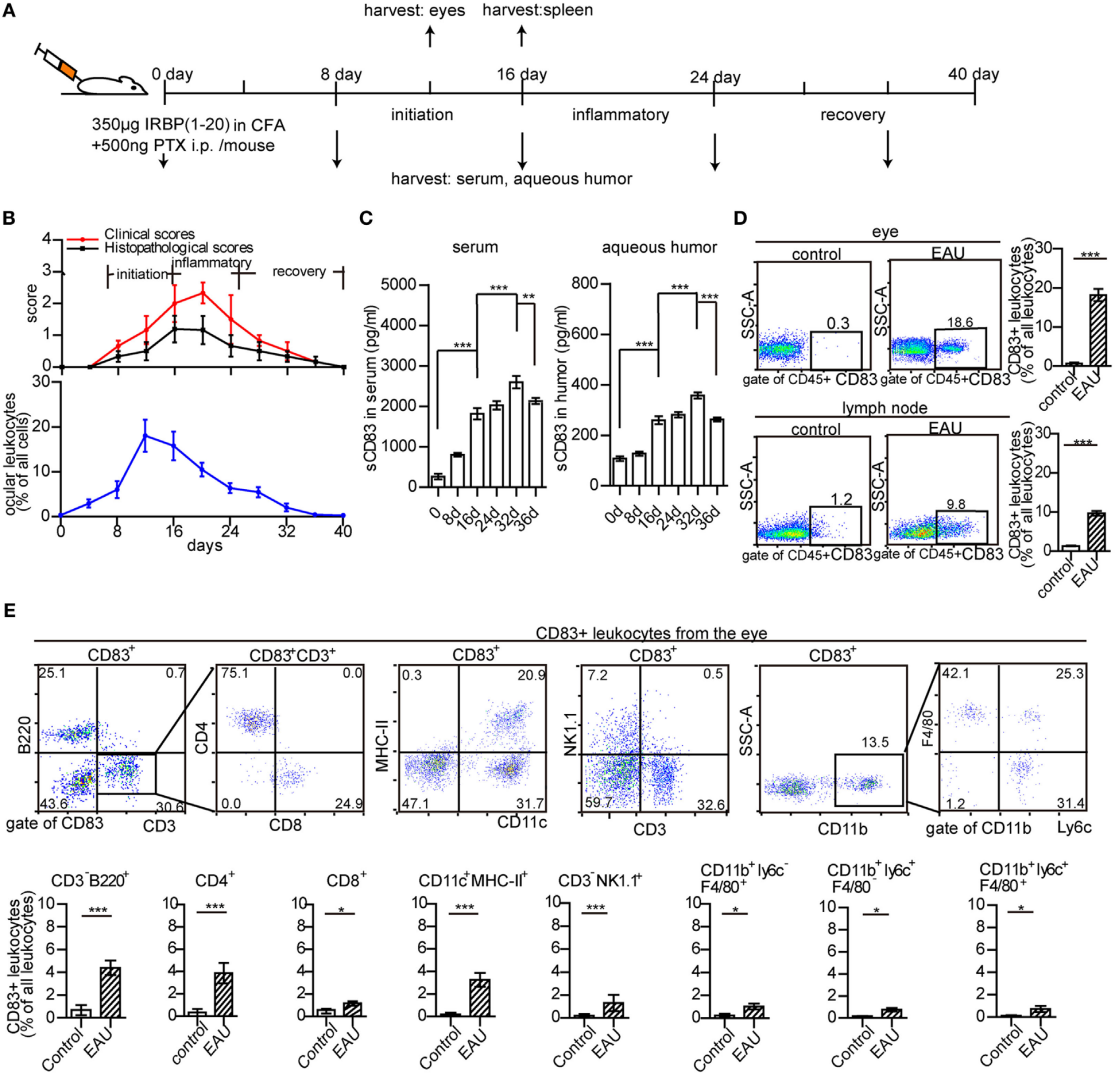
Ocular leukocyte infiltration during experimental autoimmune uveitis (EAU). **(A)** Protocol of EAU induction. **(B)** Clinical and histopathological severity score, and leukocyte infiltration in the eyes of EAU mice. **(C)** The concentration of soluble CD83 (sCD83) in the serum and aqueous humor. Data are shown as mean ± SEM, *n* = 15 in triplicates, one-way ANOVA LSD-*t* test. ***p < 0.001. **(D)** CD83^+^ leukocytes in inflamed eyes and drainage lymph node on day 12 post-immunization compared to controls. Gated on CD45^+^ cells were shown in Figure S1 in Supplementary Material. Data shown as mean ± SEM, *n* = 10 in triplicates, one-way ANOVA LSD-*t* test. ***p < 0.001. **(E)** Flow cytometry analysis of CD83 expression in different leukocyte subsets of the inflamed eye during EAU. Mean ± SEM, *n* = 10 in triplicates, two-tailed Student’s *t*-test. ***p < 0.001, **p < 0.01, *p < 0.05.

Murine EAU was induced by immunization with the peptide IRBP_1–20_ (a specific antigen for uveitis) and PTX as previously reported (40, 41) (Figure 1A). Clinical symptoms occurred on day 8, maximum severity on days 16–24, and remission from day 28 onward (Figure 1B). The peak of leukocyte infiltration in the eyes occurred between days 12 and 16 (Figure 1B; Figure S1 in Supplementary Material). It mostly comprised CD3^+^ T cells (6.9 ± 2.9% in the whole eye), CD11c^+^ MHC-II^+^ DCs (2.3 ± 1.2%), and CD3^−^ NK1.1^+^ cells (2.2 ± 1.1%), but also cells of the myeloid compartment (Figure S2A in Supplementary Material). CD3^+^ T cells, CD11c^+^ MHC-II^+^ DCs, and CD3^−^ NK1.1^+^ cells were also increased in the spleen of EAU mice (Figure S2B in Supplementary Material).

Furthermore, the dynamic change of soluble CD83 molecule (sCD83) in the serum and aqueous humor of EAU mice was analyzed. sCD83 was highly elevated on day 16, with a significant further increase on day 32 in both serum and aqueous humor (Figure 1C), suggesting a role of sCD83 in the onset and recovery phase of EAU. CD83^+^ leukocytes were absent in the eyes of control mice, while 18.6 ± 3.2% of CD45^+^ infiltrating leukocytes were CD83 positive in EAU conditions, including CD4^+^ T cells, B cells, DCs, and NK cells (Figures 1D,E). CD83^+^ leukocytes in the inflamed spleen and draining lymph nodes also increased from 10.1 ± 3.8 to 37.6 ± 4.1% and 1.2 ± 0.3 to 9.8 ± 1.6%, respectively (Figure 1D; Figures S3A,B and S4 in Supplementary Material).

### sCD83 Treatment Ameliorates EAU

**Figure 2:**
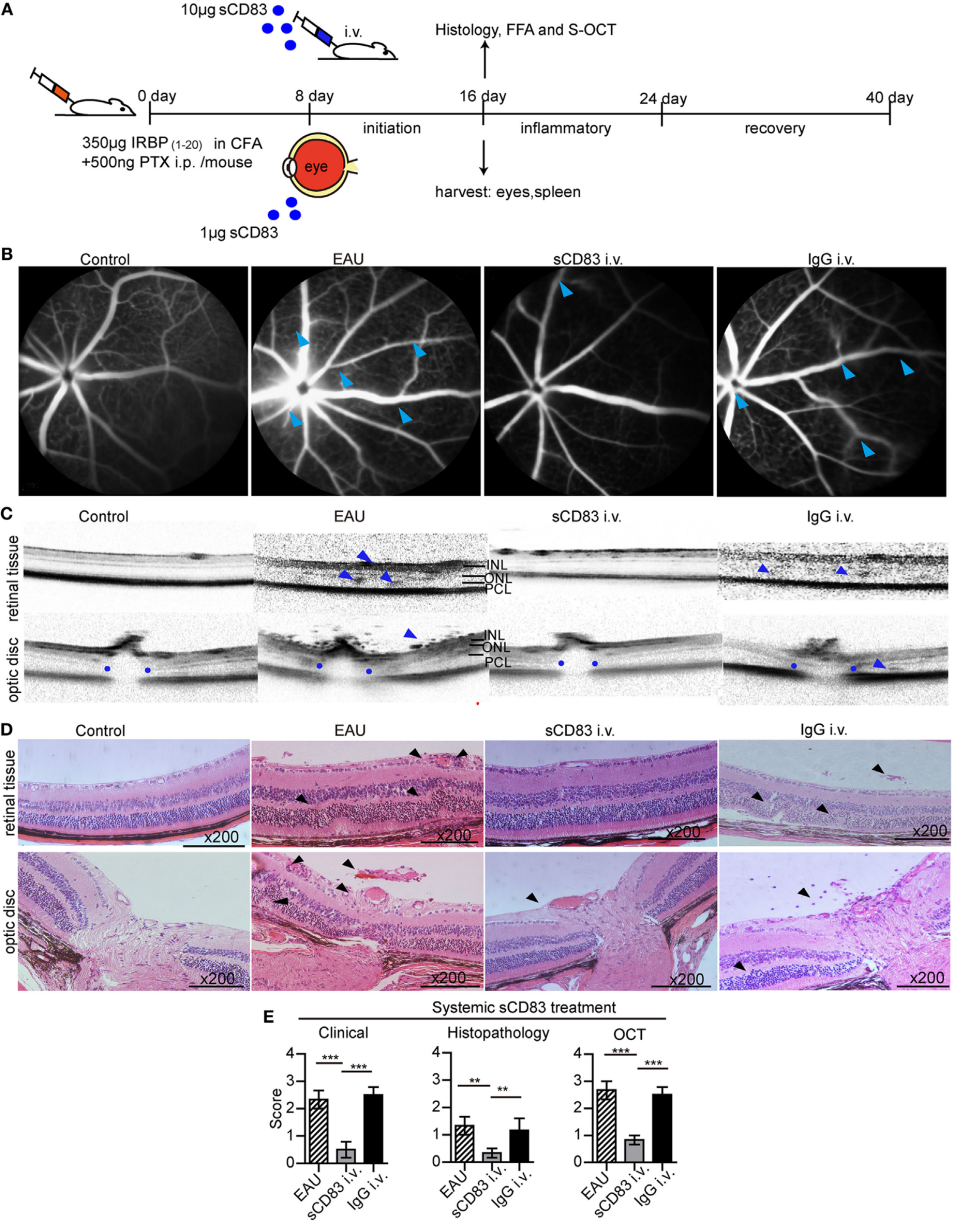
Soluble CD83 (sCD83) treatment ameliorates experimental autoimmune uveitis (EAU). **(A)** Protocol of the sCD83 treatment scheme during EAU. **(B–D)** Representative images from a control, EAU, i.v. sCD83-treated, and i.v. IgG_1_-treated mouse as assessed by **(B)** fundus fluorescein angiography, **(C)** spectralis optical coherence tomographic (S-OCT) scans, and **(D)** histology. Leaks and lacks of hyperfluorescence at the optic disk are marked [blue arrows in **(B)**]. Dark blue arrows in **(C)**, point to dome-shaped signals at the subretinal space. Blue dots point to the boundary of the outer plexiform layer of the optic disk. INL, inner nuclear layer; ONL, outer nuclear layer; PCL, photoreceptor cell layer. **(D)** H&E stainings of the retina at 200× magnification. Black arrows mark swollen blood vessels, infiltrating lymphocytes, and retinal disorganization. Scale bar = 100 μm. **(E)** The histopathological, clinical, and OCT scores were evaluated in EAU, sCD83-treated, and IgG_1_-treated mice. Mean ± SEM, *n* = 3 in triplicates, one-way ANOVA LSD-*t* test. ***p < 0.001, **p < 0.01.

To investigate the role of sCD83, we tested the impact of sCD83 on the development of EAU. After EAU induction, sCD83-IgG_1_ was injected intravenously every other day starting on day 8 (Figure 2A). Human IgG_1_-Fc was used as negative control. In mock-treated EAU mice, optic disk swelling and vascular leakage were found by FFA and OCT on day 16–21 (peak of EAU severity), indicating inflammatory infiltration, multifocal retinal disorganization, and vascular barrier breakdown (Figures 2B,C; Figure S5 in Supplementary Material). Histology showed multifocal chorioretinal lesions, severe vasculitis, and abundant lymphocyte infiltration in the vitreous compartment (Figure 2D). Fundoscopy confirmed multifocal chorioretinal defects and vasodilatation in EAU mice (Figure S6 in Supplementary Material). By contrast, intravenous sCD83 injections antagonized these changes, and only little retinal tissue damage was detectable using FFA, OCT, fundoscopy, and histology (Figures 2B–D; Figure S6 in Supplementary Material). Dye leakage, optic disk swelling, and vascular proliferation was greatly improved (Figures 2B–D). The clinical scores of eye inflammation were significantly decreased in sCD83-treated mice compared to controls (Figure 2E). Increasing amounts of administered sCD83 led to a higher reduction of inflammation (Figure S7A in Supplementary Material). However, high concentrations of sCD83 increased mortality (Figure S7B in Supplementary Material). 10 µg/mouse of sCD83 showed the best therapeutic result without detrimental side effects (Figure S7B in Supplementary Material).

Similar to intravenous injections, sCD83 eye drops also ameliorated the symptoms of vasculitis and optic disk swelling. Retinal disorganization mostly disappeared, and the histological and clinical scores of the inflamed eyes decreased (Figure S8 in Supplementary Material). Thus, systemic and topical sCD83 treatment exerted a protective effect in EAU. Notably, after sCD83 treatment for 8 days, the concentration of sCD83 in the serum of i.v. treated mice was higher than in mice with topical administration (Figure S9 in Supplementary Material).

### sCD83 Reduces Inflammatory Leukocyte Infiltration in EAU

**Figure 3:**
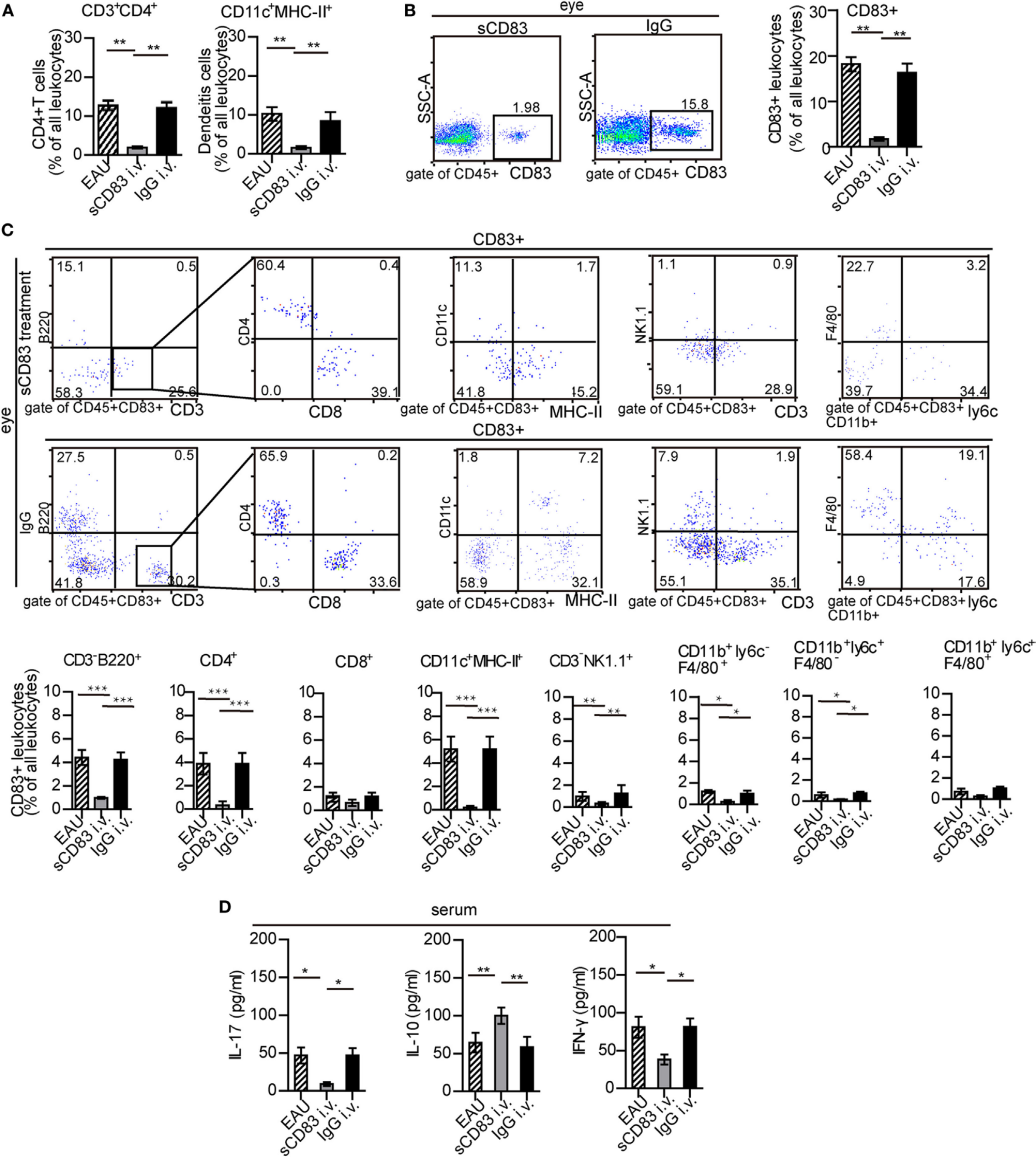
Soluble CD83 (sCD83) impedes ocular leukocyte infiltration in experimental autoimmune uveitis (EAU). **(A,B)** Flow cytometry analysis of ocular CD3^+^ CD4^+^ T cells, CD11c^+^ MHC-II^+^ dendritic cells (DCs), and CD83^+^ leukocytes from i.v. sCD83-treated mice and IgG_1_-treated mice. Gating strategy is shown in Figure S1 in Supplementary Material. **(C)** Ocular CD83^+^ leukocytes subsets were assessed by flow cytometry. **(D)** Serum concentrations of IL-17, IFN-γ, and IL-10 were determined by ELISA. All data are shown as mean ± SEM, *n* = 3 in triplicates, one-way ANOVA LSD-*t* test. ***p < 0.001, **p < 0.01, *p < 0.05.

Intravenous sCD83 treatment leads to a significant decrease of all leukocyte populations in the eyes compared to IgG_1_ controls, including CD11c^+^ MHC-II^+^ DC and CD4^+^ T cells (Figure 3A; Figure S10 in Supplementary Material). A similar effect could be observed in the spleen (Figure S10B in Supplementary Material). Topical application as eye drops caused a similar block of leukocyte recruitment in both the eye and the spleen. The reduction in cellular infiltration also included CD83^+^ leukocytes (Figure 3B). In particular, CD83^+^ B cells, CD83^+^ CD4^+^ T cells, CD83^+^ DCs, and CD83^+^ macrophages were decreased significantly (Figure 3C). Likewise, sCD83 treatment also reduced CD83^+^ cells in the inflamed spleen and lymph node (Figures S4 and S11 in Supplementary Material). By contrast, while the percentage of CD83^+^ CD4^+^ and CD83^+^ CD8^+^ T cells did not significantly change in the inflamed spleen (Figure S11 in Supplementary Material), the absolute quantity decreased. In this line, the concentrations of the cytokines IL-17 and IFN-γ in the serum decreased after sCD83 treatment, while the level of IL-10 increased (Figure 3D), suggesting an anti-inflammatory signature of sCD83 in EAU.

### sCD83 Indirectly Limits CD4^+^ T Cell Activation and Proliferation

**Figure 4:**
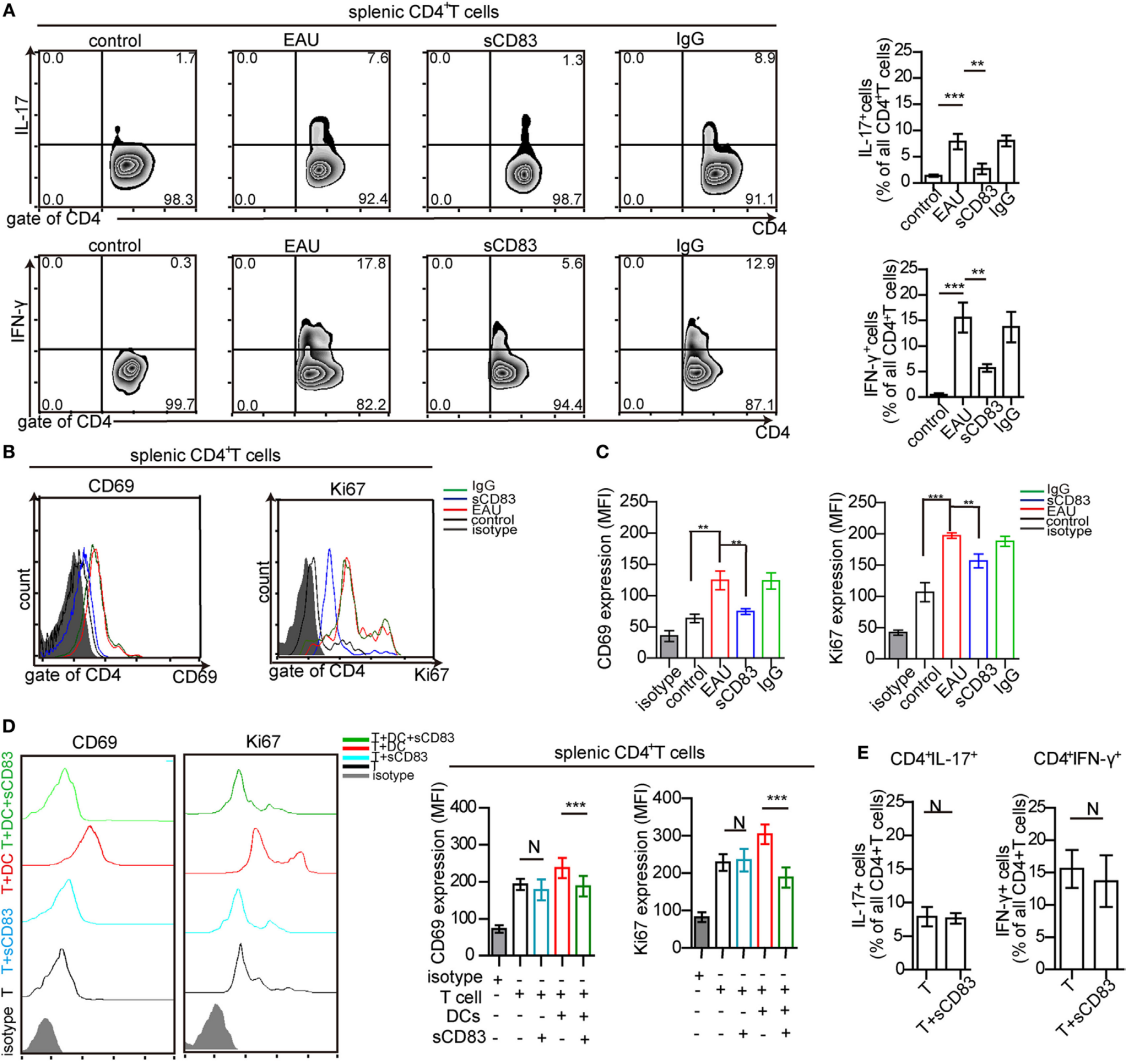
Soluble CD83 (sCD83) inhibits CD4^+^ T cell activation *in vivo* but not *in vitro*. **(A)** Abundance of CD4^+^ IL-17^+^ (type 17 helper T cells) and CD4^+^ IFN-γ^+^ (type 1 helper T cells) cells in the spleen as determined by flow cytometry in experimental autoimmune uveitis (EAU), i.v. sCD83-treated mice, IgG_1_-treated mice, and control mice. **(B,C)** The expression of CD69 and Ki67 in splenic CD4^+^ T cells **(B)** and the mean fluorescence intensity (MFI) **(C)** were analyzed by flow cytometry. Data shown in **(A–C)** are from *n* = 20 in triplicates. Mean ± SEM, one-way ANOVA LSD-*t* test. ***p < 0.001, **p < 0.01. **(D)** CD69 and Ki67 expression of splenic CD4^+^ T cells in co-culture experiments with different conditions: splenic CD4^+^ T cells of EAU mice (T), T cells with sCD83 treatment (T + sCD83), co-culture of T cells with interphotoreceptor retinoid-binding protein peptide_1–20_ (IRBP_1–20_)-pulsed mature dendritic cell (DC) (T + DC), and co-culture of T cells with sCD83-pretreated IRBP_1–20_-pulsed mature DC (T + DC + sCD83). The MFI of CD69 and Ki67 in these T cells is shown in the right histograms. *n* = 5, N: not significant, one-way ANOVA LSD-*t* test. **(E)** Flow cytometry of CD4^+^ IL-17^+^ and CD4^+^ IFN-γ^+^ T cells (% of total CD4^+^ T cells) with and without sCD83 treatment. *n* = 5, mean ± SEM, N: no significant, two-tailed Student’s *t*-test. ***p < 0.001.

Uveitogenic CD4^+^ T cells including CD4^+^ IFN-γ^+^ Th1 cells and CD4^+^ IL-17^+^ Th17 cells are crucial effectors that drive inflammation and tissue damage (7, 8). Thus, the effect of sCD83 on CD4^+^ T-cell activation and proliferation was analyzed. The CD4^+^ IFN-γ^+^ and CD4^+^ IL-17^+^ T cell subsets were decreased in the spleen of sCD83-treated (i.v.) mice, compared with untreated or IgG_1_-treated mice (Figure 4A). The expression of CD69 and Ki67 as activation markers on CD4^+^ T cells decreased (Figures 4B,C). Intravenous sCD83 treatment caused a decrease of uveitogenic CD4^+^ T cells from 8.79 × 10^4^ to about 0.8 × 10^4^ per eye. Similar results were obtained using topical sCD83 treatment. These data indicate that sCD83 treatment limits the infiltration (or differentiation) and activation/proliferation of T cells during EAU.

We next asked whether this effect was due to direct effects of sCD83 on T cells. Splenic CD4^+^ T cells were isolated from immunized mice, and treated with 10 ng/ml sCD83 *in vitro*. After 48 h, the expression of CD69 and Ki67 was unchanged compared to untreated controls (Figure 4D). Moreover, the relative frequencies of Th1 and Th17 cells did not change after sCD83 treatment (Figure 4E). Thus, sCD83 did not directly affect the activation of CD4^+^ T cells.

### sCD83 Induces Tolerogenic DCs

**Figure 5:**
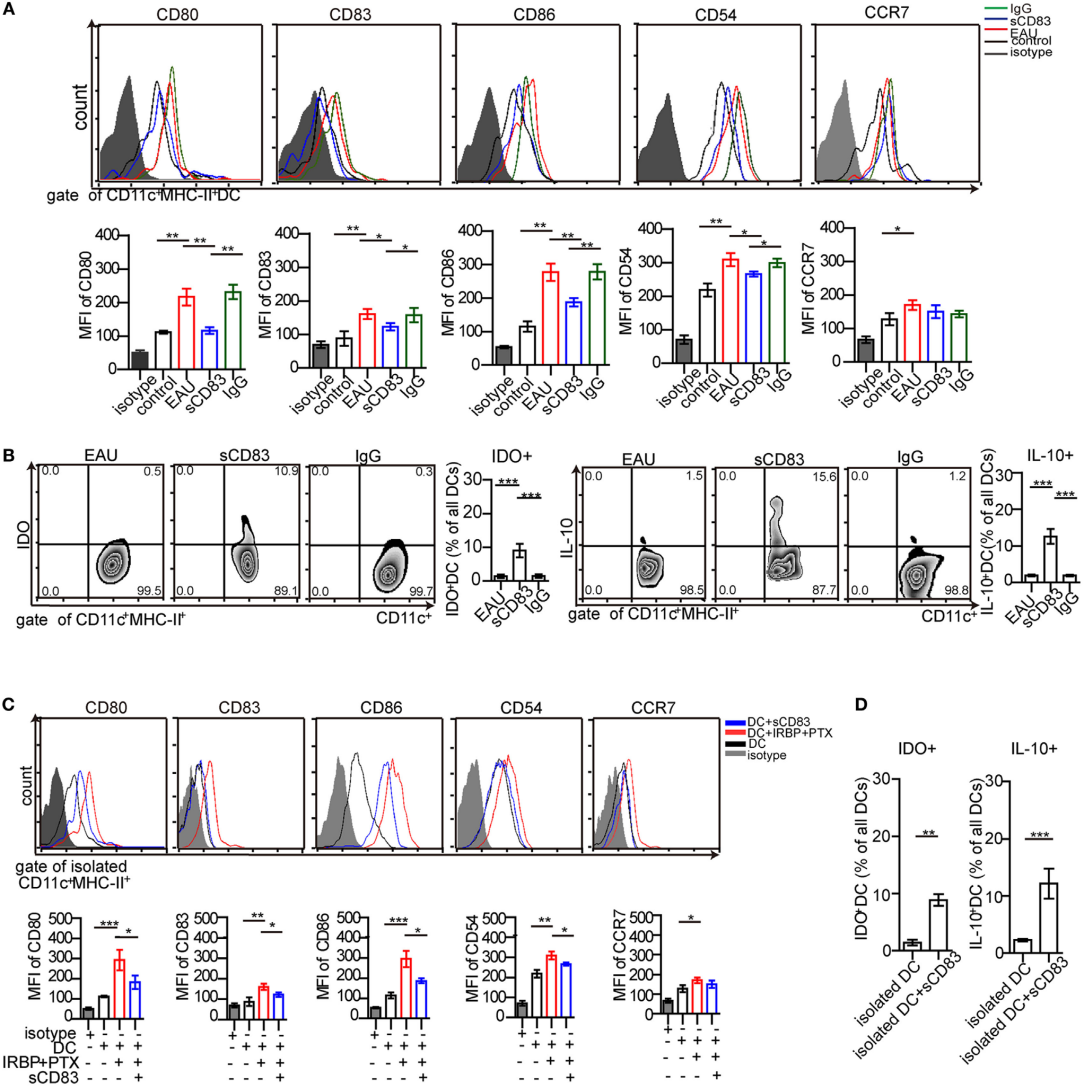
Soluble CD83 (sCD83) inhibits the maturation of dendritic cells (DCs) *in vivo* and *in vitro*. **(A)** Surface expressions of CD80, CD83, CD86, CD54, and CCR7 in splenic DCs were measured in controls, experimental autoimmune uveitis (EAU), sCD83-treated EAU, or IgG_1_-treated EAU mice. Gated on CD45^+^ CD11c^+^ MHC-II^+^ cells. **(B)** The percentage of IDO^+^ DCs and IL-10^+^ DCs from EAU mice with or without sCD83/IgG_1_ treatment. Data of **(A,B)** are from *n* = 20 in triplicates, mean ± SEM, one-way ANOVA LSD-*t* test. ***p < 0.001, **p < 0.01, *p < 0.05. **(C)** Surface expression of CD80, CD83, CD86, CD54, and CCR7 on isolated DCs were analyzed by flow cytometry *in vitro*. DCs were isolated from EAU mice (DC), matured DC pulsed interphotoreceptor retinoid-binding protein peptide_1–20_ (IRBP_1–20_) and pertussis toxin (PTX) (DC + IRBP_1–20_ + PTX), and additionally treated with sCD83 (DC + sCD83). *n* = 5, mean ± SEM, one-way ANOVA LSD-*t* test. ***p < 0.001, **p < 0.01, *p < 0.05. **(D)** Flow cytometry analysis of IDO^+^ and IL-10^+^ DCs with or without sCD83 treatment. *n* = 5; mean ± SEM, two-tailed Student’s *t*-test. ***p < 0.001, **p < 0.01.

The increased expression of CD69 and Ki67 in T cells from immunized mice in contact with DCs was abolished by sCD83 pretreatment (Figure 4D). Since DCs play a key role in uveitis by presenting antigens to CD4^+^ T cells (2), the effect of sCD83 on T cells could be caused by DCs tolerance. Then, the impact of sCD83 on DC was tested *in vivo* and *in vitro*. The surface expression of CD80, CD83, CD86, and CD54 (ICAM-1) on CD11c^+^ MHC-II^+^ DCs in EAU mice decreased after intravenous sCD83 treatment while CCR7 expression did not change (Figure 5A). The relative frequency of IDO^+^ and IL-10^+^ DCs increased after sCD83 treatment (Figure 5B), whereas the absolute quantity of DCs decreased from 1.12 × 10^4^ to 1.2 × 10^3^/eye. Similar data were obtained from topical sCD83 treatment.

Next, the effect of sCD83 on DCs was examined *in vitro*. Splenic CD11c^+^ MHC-II^+^ DCs from EAU were isolated. After sCD83 treatment (10 ng/ml) for 24 h, DCs showed decreased expression of CD80, CD83, CD86, and CD54 (Figure 5C). In addition, IDO and IL-10 in CD11c^+^ DCs were upregulated (Figure 5D). These data indicate that sCD83 limits the maturation of DCs and induces the tolerogenic status of DCs.

### sCD83 Modulates the Calcium Response in DCs

**Figure 6:**
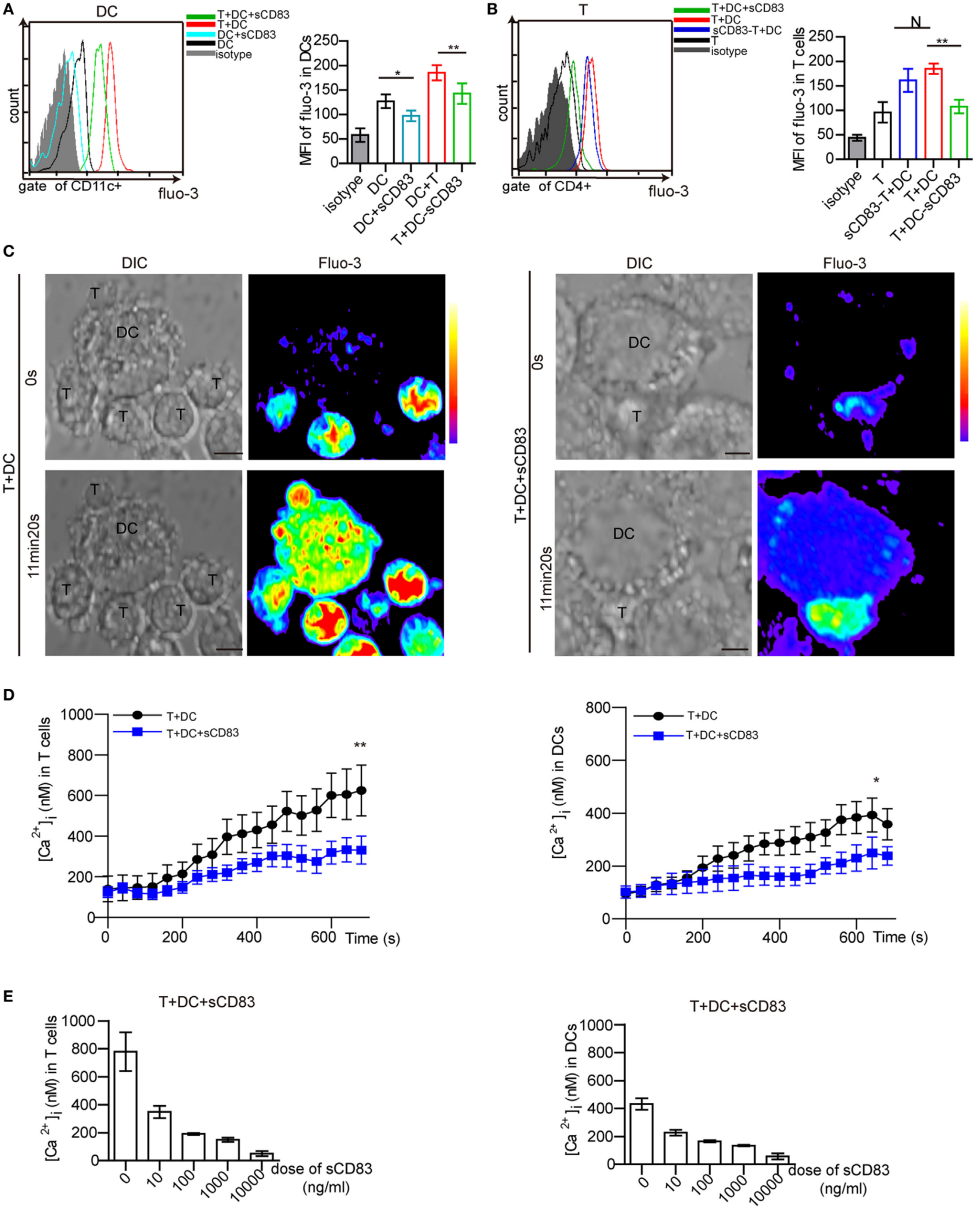
Soluble CD83 (sCD83) suppresses the calcium release in dendritic cells (DCs) interacting with CD4^+^ T cells. **(A)** Flow cytometry was used to analyze the calcium signal in DCs compared with sCD83-pretreated DCs, DCs in contact with CD4^+^ T cells, and sCD83-pretreated DCs in contact with CD4^+^ T cells. **(B)** The calcium signal in CD4^+^ T cells, sCD83-pretreated T cells in contact with DCs, T cells in contact with DCs or sCD83-pretreated DCs. Data of **(A,B)** are from three separate experiments; mean ± SEM, N: not significant, one-way ANOVA LSD-*t* test. **p < 0.01, *p < 0.05. **(C)** Live cell imaging of the calcium release in DC2.4 cells (±sCD83 treatment) in co-culture with CD4^+^ T cells by confocal microscopy. Time-lapse scanning was performed with a 40 s acquisition interval, and sustained for 10 min. Scale bar = 5 µm. Fluorescence intensity increased from lower to top (right color bar). **(D)** The dynamic calcium signal over time in CD4^+^ T cells in contact with DCs or sCD83-treated DCs (left) and DCs (±sCD83 treatment) (right) are shown during the immunological synapse. *n* = 30 in triplicates, mean ± SEM, Mann–Whitney *U* tests. **p < 0.01, *p < 0.05. **(E)** Dose-dependent effect of sCD83 on the calcium signal in CD4^+^ T cells (left) in contact with sCD83-treated DCs and DCs (right) in contact with T cells. Data are from three separate experiments, 15 cells were measured for every group.

Calcium release is a proximal signaling response during cell activation, and is required for the activation and maturation of DCs (29, 30). By flow cytometry analysis, we found that sCD83-treated DCs showed lower levels of calcium signals than untreated controls (Figure 6A). Moreover, sCD83-treated DCs in contact with T cells also released lower calcium levels than untreated DCs (Figure 6A). We also found a reduced calcium release in CD4^+^ T cells that engaged with sCD83-treated DCs, but not with untreated DCs (Figure 6B). By contrast, this effect was not evident in sCD83-treated T cells engaging with DCs (Figure 6B). These experiments suggest that sCD83 predominantly targets DCs and impairs calcium signaling.

Next, the T cell–DC synapse was investigated by live cell imaging *in vitro*. DC2.4, a mouse DC line, has been shown to have a similar phenotype as *in vivo* derived DCs (36, 44, 45). Maturation of DC2.4 is induced by IRBP_1–20_ and PTX, which can be blocked by sCD83 treatment (Figure S12 in Supplementary Material). Isolated CD4^+^ T cells were co-cultured with matured DC2.4 cells. While stable synapses were observed in untreated conditions, the addition of sCD83 decreased the percentage of T–DCs contacts (Figure S13 in Supplementary Material). A stable synapse triggered a fast and high level of calcium release in both CD4^+^ T cells and DC2.4 cells (Figure 6C, left). By contrast, sCD83-treated DC2.4 cells showed a low level of calcium release in both DC2.4 cells and contacting CD4^+^ T cells (Figure 6C, right). Moreover, the peak calcium signal in T cells contacting sCD83-treated DC2.4 cells was lower than in controls (untreated DC2.4 cells) (Figure 6D, left). sCD83 treatment also decreased the peak calcium signaling in DC2.4 cells (Figure 6D, right). The blocking effect of sCD83 on the calcium release was concentration dependent (Figure 6E). Together, these imaging data confirm that sCD83 exerts a blocking effect on DC activation that subsequently results in impaired CD4^+^ T-cell activation.

### sCD83 Affects the Spatial Localization of Calcium Microdomains

**Figure 7:**
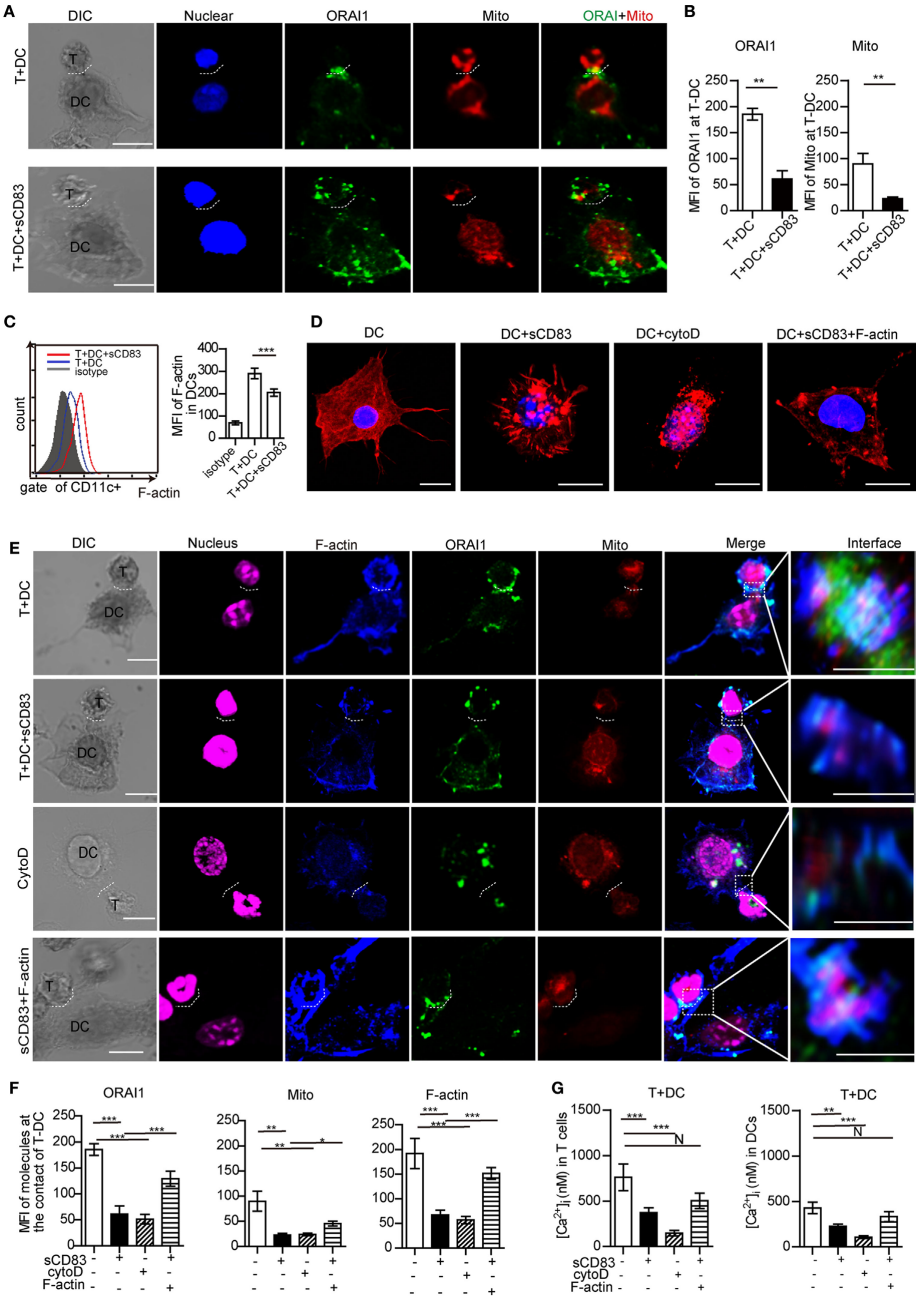
Soluble CD83 (sCD83) disrupts cytoskeletal filamentous actin (F-actin) and the topology of calcium microdomains in antigen-presenting dendritic cells (DCs). The localization of molecules on T cells and DCs was analyzed by confocal microscopy. T–DC doublets were chosen from bright field images and evaluated using fluorescence image stacks. **(A)** Localization of ORAI1 (green) and mitochondria (red) at the contact zone of sCD83-treated DC2.4-T cells and untreated DC2.4-T cells. The dotted lines mark the synapse of DC2.4-T. Scale bar = 5 µm. **(B)** The mean fluorescence intensity (MFI) of ORAI1 or mitochondria at the contact zone of T cell–DC interaction. Mean ± SEM, 15 cell-contacts were measured for every group from three independent experiments, two-tailed Student’s *t*-test. **p < 0.01. **(C)** The expression of F-actin in DCs in contact with T cells (red) and sCD83-pretreated DCs co-cultured with T cells (blue) was analyzed by flow cytometry (isotype: gray). Mean ± SEM, *n* = 15 cells from three independent experiments, two-tailed Student’s *t*-test. ***p < 0.001. **(D)** The distribution of F-actin (red) in DCs, sCD83-pretreated DCs, cytochalasin D-pretreated DCs, and sCD83-pretreated DC2.4 with F-actin overexpression. Scale bar = 5 µm. **(E)** Co-localization analysis of F-actin (blue), ORAI1 (green), and mitochondria (red) at the DC2.4-T cell synapsis, and the effect of sCD83, cytochalasin D, and F-actin overexpression. The dotted lines mark the DC–T synapse. Scale bar = 5 µm. The right panels show the enlarged view of the contact zone. A three-dimensional image of DC–T contact was reconstructed. Scale bar = 2 µm. **(F)** Quantification of the MFI of ORAI1, mitochondria, and F-actin at the contact zone in different conditions. **(G)** The mean calcium concentration in contacting T cells and DCs was assessed. Data of **(F,G)** are shown as mean ± SEM, *n* = 15 for every group from three independent experiments. One-way ANOVA LSD-*t* test. N: no significant, ***p < 0.001, **p < 0.01, *p < 0.05.

Next, we determined the effect of sCD83 on the localization of ORAI1 and mitochondria at the contact of DC2.4 and CD4^+^ T cells. In untreated conditions, ORAI1 was localized at the T–DCs synapse (Figures 7A,B; Figure S14A in Supplementary Material). In sCD83-treated conditions, ORAI1 failed to accumulate at the contact of T–DCs (Figures 7A,B; Figure S14A in Supplementary Material). Moreover, mitochondria formed aggregates at the contact of DC and T cells in untreated conditions, which was not observed after sCD83 pretreatment of DCs (Figures 7A,B; Figure S14A in Supplementary Material). These observations indicate that a disruption of calcium microdomain kinetics in DCs underlies the defective calcium signaling mediated by sCD83.

### sCD83 Disrupts F-actin Accumulation Required for the Calcium Response

As cytoskeletal F-actin critically regulates the calcium release (31, 36), we analyzed the expression of F-actin in DCs with or without sCD83 treatment. Indeed, sCD83 caused a decreased expression of F-actin in DCs compared to untreated controls (Figure 7C). After sCD83 treatment, DCs became rounded and showed only short and truncated, or no protrusions at all (Figure 7D; Figure S14B in Supplementary Material). Similar morphological changes were observed after F-actin depolymerization with cytochalasin D (Figure 7D; Figure S14B in Supplementary Material). Furthermore, F-actin lost the capability to accumulate at the contact of DC and T cells in sCD83-treated conditions (Figures 7E,F; Figure S14A in Supplementary Material). Cytochalasin D-mediated disruption of the cytoskeleton abolished almost all T–DCs interaction, and no calcium signaling was detected in DCs (Figures 7E,G; Figure S14 in Supplementary Material). Overexpression of F-actin in sCD83-treated DCs could rescue this phenotype, including a normal cellular morphology with multiple dendrites and a normal calcium release response (Figures 7D,G).

Filamentous actin co-localized with ORAI1 and mitochondria at T–DC contact (Figure 7E). sCD83 treatment reduced the accumulation of F-actin at the T–DCs contact and caused significantly lower expression of ORAI1 and mitochondria (Figures 7E,F; Figure S14A in Supplementary Material). Similar to cytochalasin D, sCD83 abrogated F-actin co-localization with ORAI1 and mitochondria (Figure 7E; Figure S14A in Supplementary Material). Overexpression of F-actin in sCD83-treated DCs again rescued this phenotype (Figure 7E). Together, these results indicate that F-actin disruption was a main effector mechanism of sCD83 in DCs that leads to impaired dynamic positioning of calcium microdomains and calcium release upon T–DCs contact.

### sCD83 Decreases the Synaptic Accumulation of CD54 at the DC–T Cell Contact

**Figure 8:**
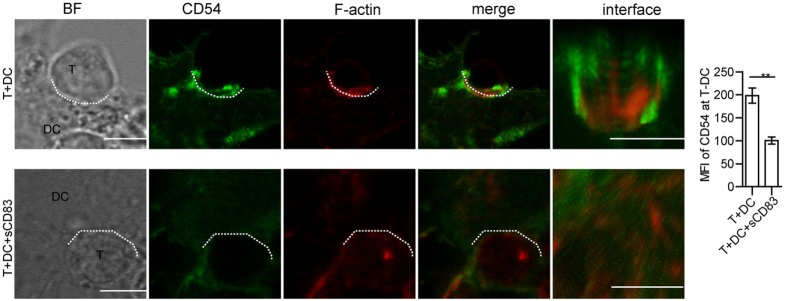
Soluble CD83 (sCD83) affects the accumulation of CD54 at the contact zone of dendritic cell (DC) and T cells. The localization of CD54 (green) and filamentous actin (F-actin) (red) at the DCs–T contact zone was analyzed by confocal microscopy with untreated (top row) or sCD83-treated DC2.4 (bottom row). The dotted lines mark the DCs–T synapse. Scale bar = 5 µm. Mean ± SEM *n* = 15 for every group from three independent experiments, two-tailed Student’s *t*-test. **p < 0.01.

Synaptic molecules such as CD54 (ICAM-1) are regulated by cytoskeletal F-actin, and the localization of CD54 at the T–DC contact zone correlated with T cell activation (45, 46). To further investigate the mechanism of how sCD83-treated DCs fail to induce CD4^+^ T cell activation, we measured the localization of CD54. In untreated conditions, CD54 co-localized with F-actin at the synapse, whereas after sCD83 treatment this molecular distribution was abrogated (Figure 8; Figure S14C in Supplementary Material). Similarly, with sCD83 treatment, CD80 and CD86 also failed to accumulate at the synapse (Figure S15 in Supplementary Material). These data suggest that the sCD83-mediated disruption of the cytoskeleton affects the localization of co-stimulatory molecules on DCs during the immunological synapse.

## Discussion

Uveitis is a sight-threatening autoimmune disorder with only few approved treatment regimens (5). In this study, we describe the beneficial effect of sCD83 on autoimmune uveitis in mice, and identify an immunomodulatory role of sCD83 on DCs to further inhibit T-cell activation. Furthermore, we present a new mechanism of sCD83 that involves F-actin disruption, and affects calcium signaling and the expression of co-stimulatory surface molecules in DCs (Figure S16 in Supplementary Material). Our data imply that sCD83 could represent a novel therapeutic molecule for the treatment of human autoimmune uveitis.

Uveitis is an autoimmune disease that affects many different immune compartments (7, 8, 11, 47, 48). The level of sCD83 in both serum and aqueous humor increased during the inflammatory phase, peaked at the initial stage of recovery phase of EAU, and decreased after day 32 of EAU (Figure 1C). It indicated that a local protective response is mounted early during EAU. We hypothesize that inflammation dominates the local milieu until adoptive immunity is sufficiently shifted to a regulatory phenotype. sCD83 treatment reduces leukocyte infiltration, including CD83^+^ subsets, in both inflamed eyes and the spleen, pointing to systemic effects. As CD83 expression is associated with an activated cellular state in DCs, NK cells, T and B cells (19, 20, 22, 24, 25), sCD83 application seems to impair the recruitment of activated and harmful leukocytes to the eyes (Figure S16 in Supplementary Material). Intriguingly, topical administration as eye drops was equally effective to mount a protective response in EAU mice compared to i.v. injection. However, the mechanism of topical sCD83 treatment on EAU may be different. Topical sCD83 treatment did not increase the concentration of sCD83 in the serum of topical sCD83-treated mice, compared with IgG-treated and untreated mice, whereas systemic treatment did. sCD83 is a small molecule of 12.3 kDa and is capable of rapid tissue penetration. We hypothesize that a local immunomodulatory function is exerted in the eye. Topical sCD83 treatment might inhibit the activation of DCs or trigger IDO expression in DCs, leading to suppressed T cell activation and infiltration, and thus reduces the damage of the eyes (28, 40). However, we cannot exclude a more systemic effect since the eye is highly vascularized and drained by local lymph nodes (12, 49). The mechanism of topical sCD83 treatment will be further elaborated in the future studies.

The protective mechanism of sCD83 was related to a decrease of infiltrating CD4^+^ T cells that are believed to be main inflammatory mediators in EAU (7–11). In our experiments, sCD83 did not directly affect CD4^+^ T cells, but rather modulate the maturation and activation of CD11c^+^ DCs as antigen-presenting cells. This mechanism resembles the effects of sCD83 in experimental colitis (27). Previous reports showed that altering the maturation of DCs could contribute to the recovery of EAU (2). Tolerant DCs limited the activation of CD4^+^ T cells and increased the population of Tregs (28). In this line, we show that sCD83 treatment leads to increased IDO and IL-10 expression in DCs, suggesting a shift to Treg induction. In addition, such tolerogenic DCs also might traffic through ocular compartments to the secondary lymphoid organs to promote systemic tolerance (5, 49). Together, our data suggest that beneficial effects of sCD83 in EAU might be caused by a shift from stimulatory to tolerant DCs. Previously we reported that sCD83 could inhibit the activation of NK cells by decreasing the CD11b expression during in EAU (40). Thus, sCD83 is pleiotropic and exerts effects on both the innate and adaptive immunity.

Soluble CD83 was reported to alter signaling cascades that influence the surface molecules expression and the cytoskeletal organization in DCs (26, 50, 51). sCD83 binds to the TLR4/MD-2 complex, leading to the degradation of IL-1R-associated kinase-1 and the induction of anti-inflammatory mediators such as IDO and IL-10 (26). Herein, we report a novel pathophysiological effect of sCD83 on DCs to suppress F-actin-dependent calcium signaling by disrupting the spatial localization of ORAI1 and mitochondria at DCs–T cell synapses. The localization of ORAI1 at the cell–cell contact is required for calcium influx, and the proper re-localization of mitochondria prevents calcium-dependent channel inactivation (31–33, 36). It sustains the activity of calcium release-activated calcium channel proteins for a long period to allow for a prolonged calcium influx, suggesting that a critical mechanism of sCD83 is the altered calcium response in DCs. The exact mechanism of this pathway will be subject to further investigation.

Together, our data provide evidence for altered calcium signaling mediated by F-actin disruption as a basis for the immunomodulatory role of sCD83 on DCs during EAU. It thus establishes sCD83 as a potential new player in the treatment of autoimmune uveitis.

## Ethics Statement

This study was carried out in accordance with the Chinese guidelines for the care and use of laboratory animals. All experiments were approved by the ethics committee of Shandong Academy of Medical Sciences (Jinan, China).

## Author Contributions

WL and ZF designed research, generated the figures and tables, and drafted and revised the manuscript. KB interpreted data, and drafted and revised the manuscript. WL, BW, and HB performed animal experiments, the clinical examination, flow cytometry analysis, and imaging experiments. PL, NS, YY, BL, and CL performed cells culture, cell isolation, and imaging. All authors approved the final version of this manuscript.

## Conflict of Interest Statement

The authors declare that the research was conducted in the absence of any commercial or financial relationships that could be construed as a potential conflict of interest.
