# Supplementary material for: Soluble CD83 Alleviates Experimental Autoimmune Uveitis by Inhibiting Filamentous Actin-Dependent Calcium Release in Dendritic Cells
Source: Front Immunol. 2018 Jul 9;9:1567. doi: 10.3389/fimmu.2018.01567 (PMC6052908; doi:10.3389/fimmu.2018.01567)
Supplement: Supplementary file 1 [file data_sheet_1.PDF]

Supplemental Material

Manuscript: “Soluble CD83 alleviates experimental autoimmune uveitis by inhibiting F-actin-dependent calcium release in dendritic cells.”

Authors: Wei Lin[1,2,3]*†, Konrad Buscher[4,5]†, Beibei Wang[2], Zhichao Fan[5], Nannan Song[1], Peng Li[1], Yingying Yue[1], Bingqing Li[1], Cuiling Li[1], Hongsheng Bi[2]

Table 1 Criteria of scoring in EAU based on clinical symptoms, histopathology and SD-OCT (1, 2)

Score	Clinical scoring	Histopathological scoring	SD-OCT scoring
0	No change	No change	No abnormal image
0.5	A few small, peripheral, focal, chorioretinal lesions and/or minimal vasculitis/vitritis	Small cellular infiltrates in ciliary body, vitreous and retina, No tissue damage	
1	Mild vasculitis, a few small focal chorioretinal lesions, and/or linear chorioretinal lesions	Mild inflammatory cells in the vitreous and retina; Focal retinal folding, little retinal detachment Perivasculitis; Mild vitritis;	Few high reactive dots in the vitreous and around retinal vessels
2	Multifocal chorioretinal lesions, severe vasculitis, and/or a few linear chorioretinal lesions	Mild photoreceptor loss granulomatous infiltrates per section in retina and choroid; Retinal folding and detachment; focal photoreceptor cell damage, Vasculitis in 10% of vessels	High reactive dots in the vitreous and around retinal vessels more than grade 1; High reactive dots in the retinal all layer; Few high reactive mass in the outer retina; Partial retinal layer disruption
3	Pattern of linear chorioretinal lesions; large, confluent chorioretinal lesions; and/or subretinal hemorrhage, and/or optic nerve blurring	Multiple granulomatous infiltrates; Vasculitis in 20-50% of vessels; Extensive retinal folding with detachment; serous exudates and subretinal bleeding, moderate photoreceptor cell damage ; Medium vitritis	Many high reactive dots in the vitreous, around vessels, and in all retinal layers; Multiple high reactive mass in the outer retina; Disturbance of the total retinal layer structures; Disappearance of the integrities of the external limiting membrane and inner segment/outer segment junction
4	Marked retinal detachment and/or retinal atrophy	Severe photoreceptor loss; Vasculitis in > 50% of vessels ; Severe vitritis	Disappearance of the outer retinal layers

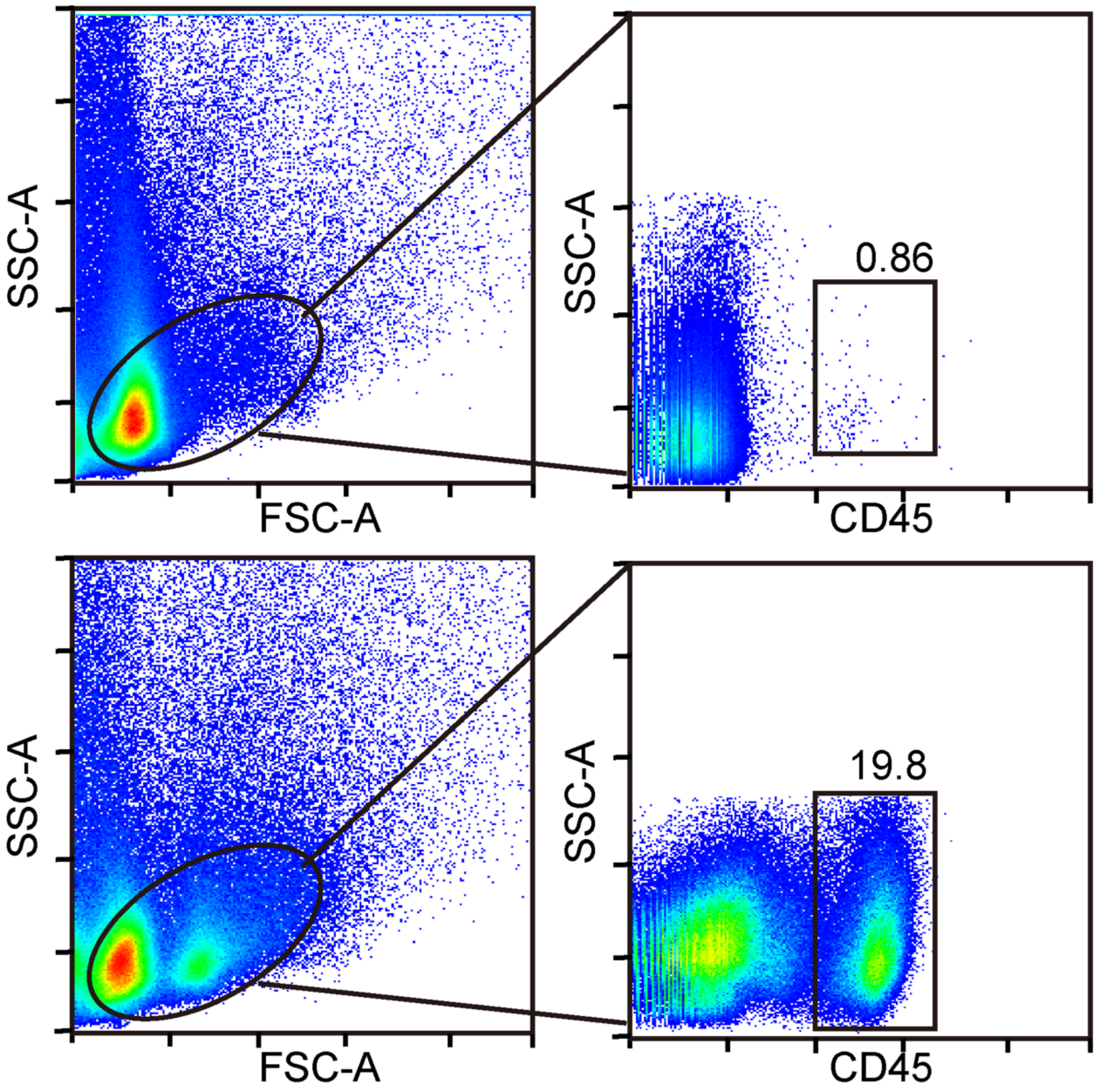

Supplemental Figure 1: Gating of ocular leukocytes. Eyes were collected at 0 days (up pannel) or 16 days post-immunization (bottom pannel) and eye-infiltrating cells were analyzed by flow cytometry. The gate of ocular CD45$^+$ leukocytes is shown.

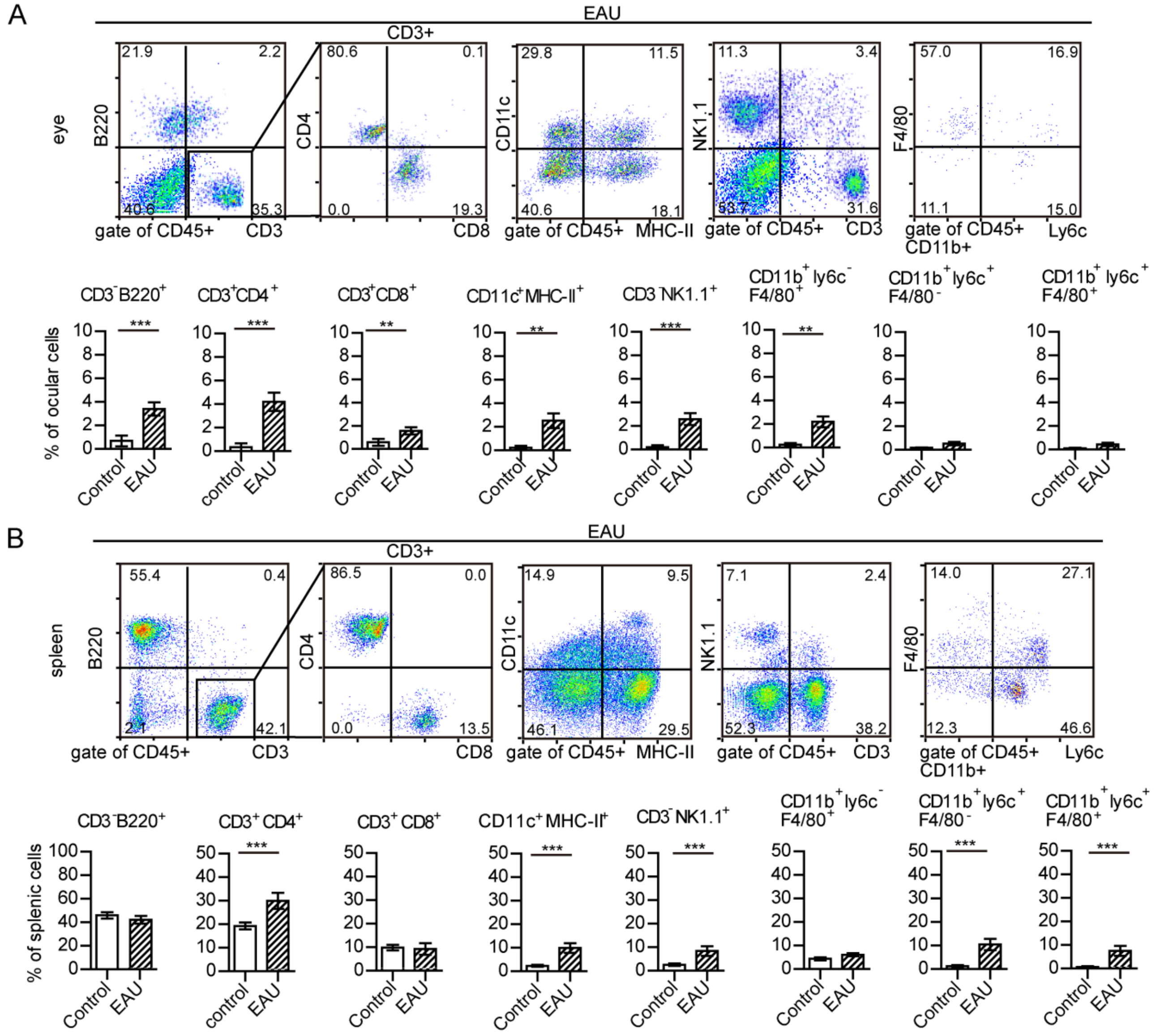

Supplemental Figure 2: The leukocyte landscape in the eye and spleen during EAU. Leukocyte subsets in the eye **(A)** and spleen **(B)** from EAU and controls. Representative flow cytometry graphs and aggregated data were from n = 10 in triplicates are shown. Mean ± s.e.m., two-tailed Student's *t* test. **p*<0.05, ** *p*<0.01, and *** *p*<0.001.

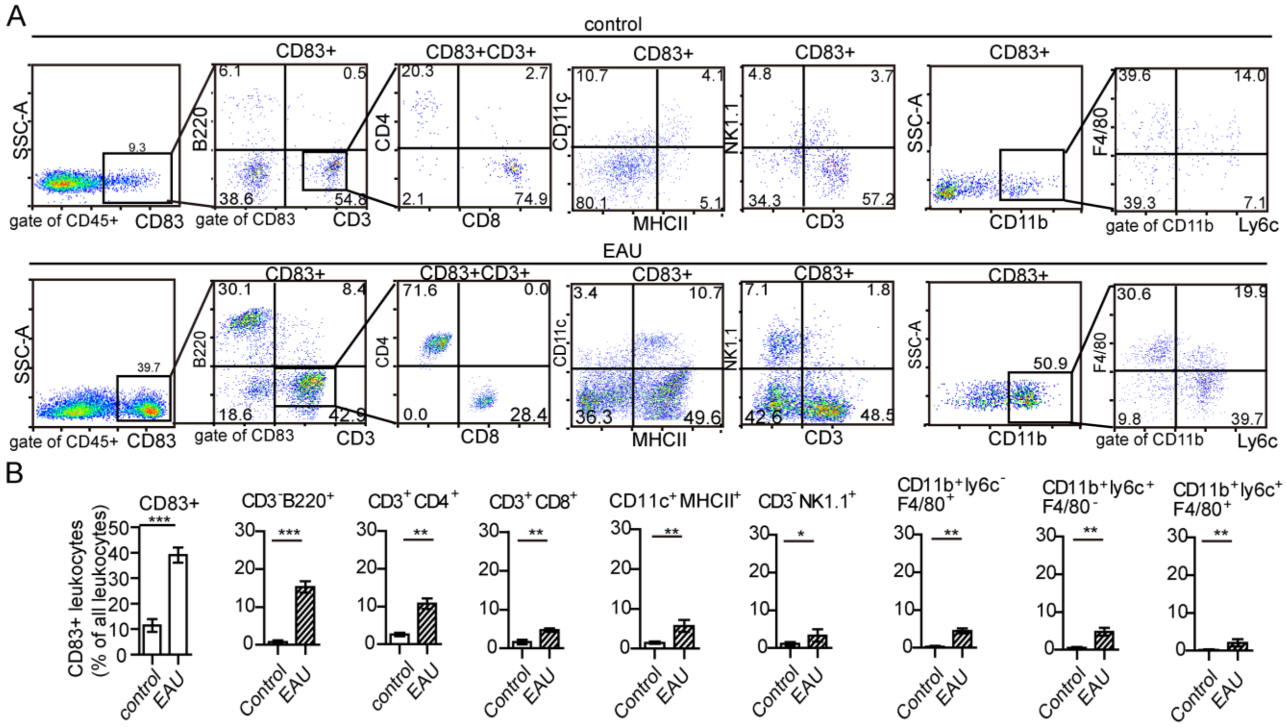

Supplemental Figure 3: CD83$^+$ leukocytes in the spleen during EAU. (A) Representative flow cytometry graphs and **(B)** aggregated data of CD83$^+$ leukocytes in the spleen from EAU mice and controls are shown. Mean ± s.e.m., n = 10 in triplicates, two-tailed Student's *t* test. **p*<0.05, ** *p*<0.01, and *** *p*<0.001.

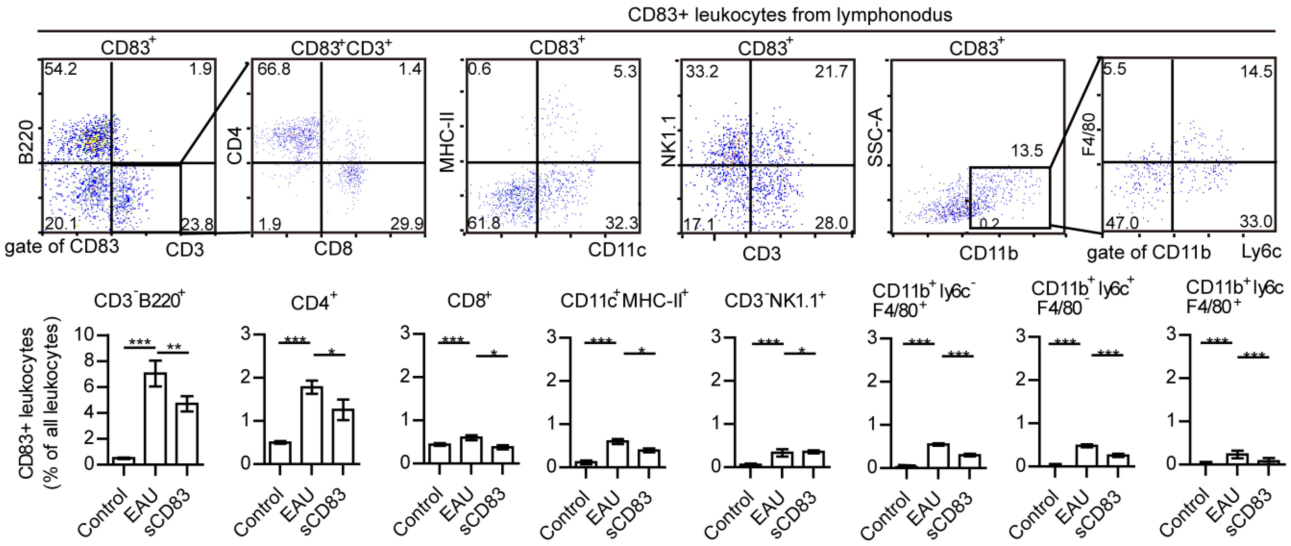

Supplemental Figure 4: Flow cytometry analysis of CD83 expression in different leukocyte subsets of the draining lymph nodes during EAU. Mean ± s.e.m., n = 10 in triplicates, Two-tailed Student's *t* test. *** *p*<0.001, ***p*<0.01, * *p*<0.05.

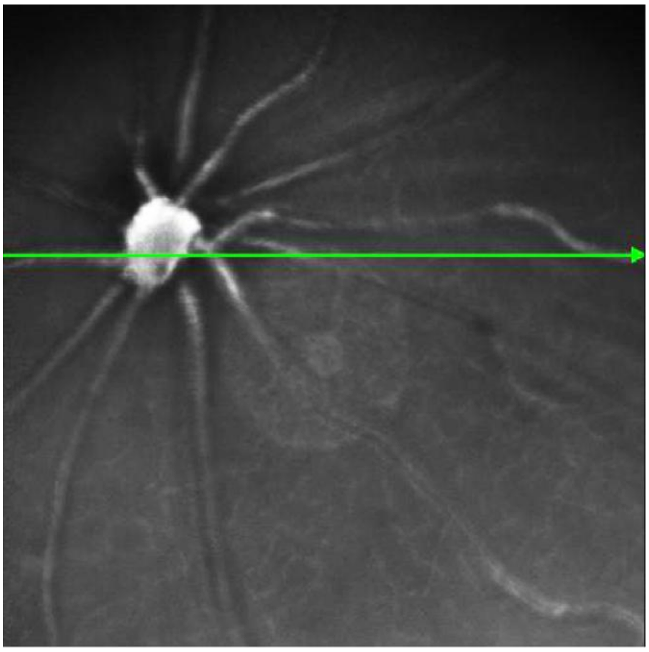

Supplemental Figure 5: The image marks the position and orientation of OCT scan. The location and orientation of the OCT scan were shown by green arrow. OCT scanning direction is along the sagittal axis of the optic disc through the green line.

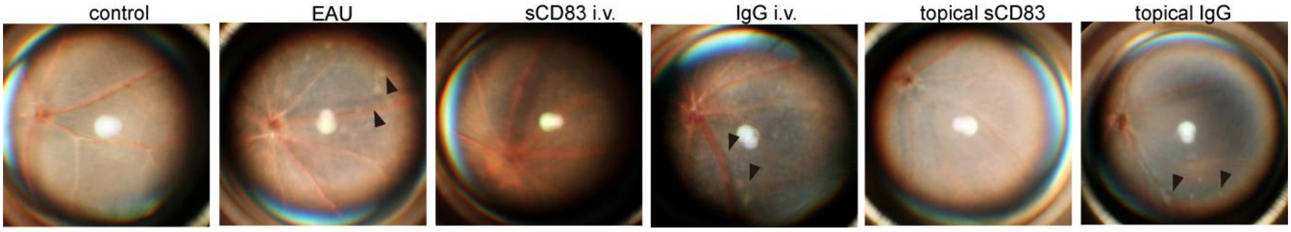

Supplemental Figure 6: Fundoscopy of EAU and sCD83 treated mice. Representative images of eyes are in different treatment conditions. Multifocal chorioretinal lesions, severe vaculities, and linear lesions were observed on the eyes of EAU mice and IgG_1 treated mice (black arrows).

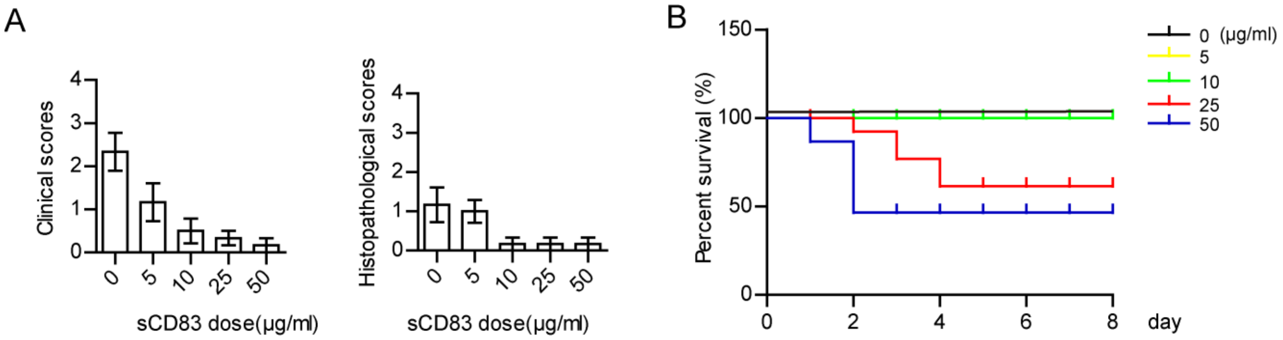

Supplemental Figure 7: Efficacy and mortality in sCD83 treatment experiments. (A) The clinical and histopathological scores, and **(B)** survival ratio of EAU mice were determined for different sCD83 concentrations (i.v. administration). Data were from three independent experiments; three mice were used for each group in an experiment, Data are shown as mean ±s.e.m.

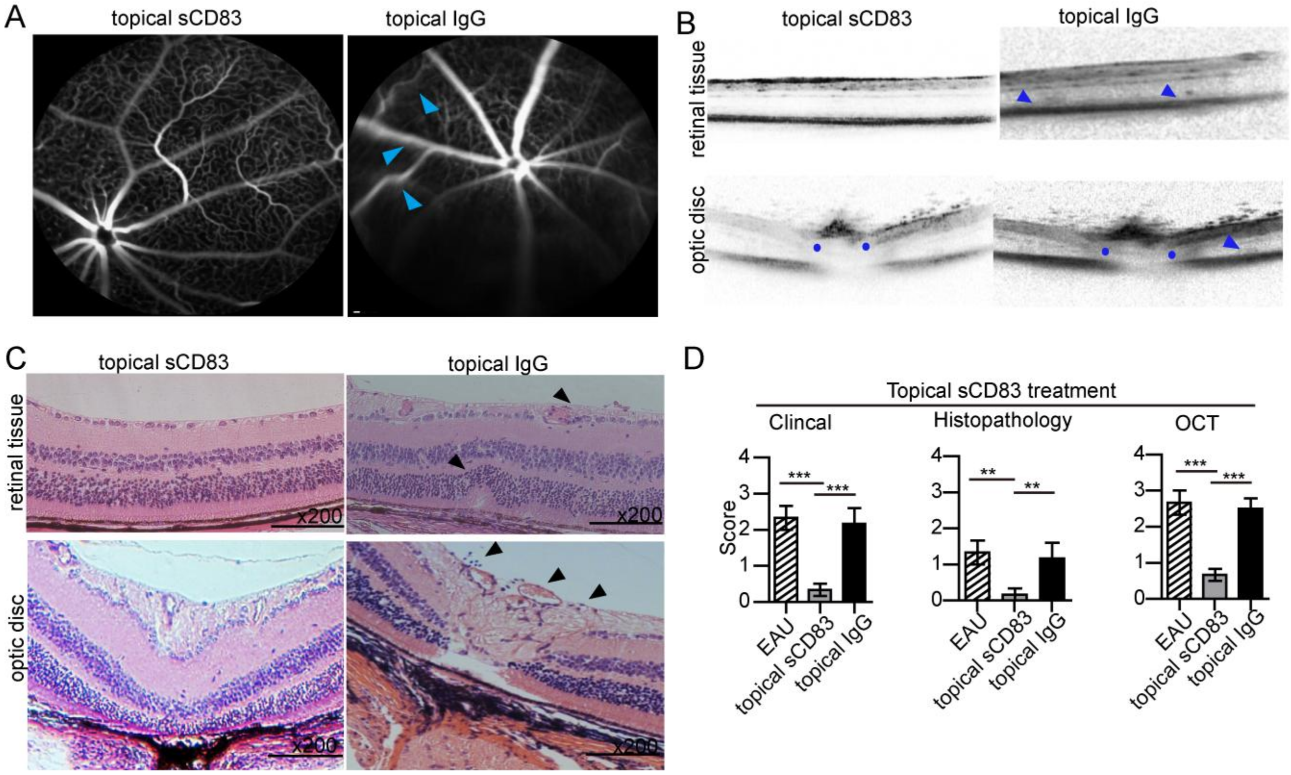

Supplemental Figure 8: Topical sCD83 treatment ameliorates EAU. (A-C) Representative images from a topical sCD83-treated, and topical IgG_1-treated mouse as assessed by **(A)** Fundus Fluorescein Angiography, **(B)** S-OCT scans and **(C)** histology. Leaks and lacks of hyperfluorescence at the optic disc are marked (blue arrows in A). Dark blue arrows in B, point to dome-shaped signals at the subretinal space. Blue dots point to the boundary of the outer plexiform layer of the optic disc.**(C)** H&E stainings of the retina at 200× magnification. Black arrows mark swollen blood vessels, infiltrating lymphocytes and retinal disorganization. Scale bar=100 µm. **(D)** The histopathological, clinical and OCT scores were evaluated in EAU, topical sCD83-treated and IgG_1 treated mice. Mean ± s.e.m., n=3 in triplicates, one-way ANOVA LSD-t test. *** p<0.001, **p<0.01.

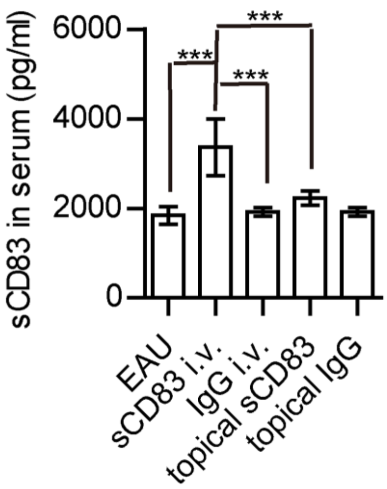

Supplementary Figure 9. Comparison of i.v. and topical administration of sCD83 on the systemic sCD83 concentration. On day 8 after immunization, sCD83 protein was either administered i.v. or topical administered every other day. The concentration of sCD83 in the serum of sCD83 i.v. treated-mice or topical sCD83 treated mice was detected at 8 days after treatment and compared with IgG-treated-mice and untreated mice. Mean ± s.e.m., n = 10 in triplicates, one-way ANOVA LSD-t test, *** $p<0.001$.

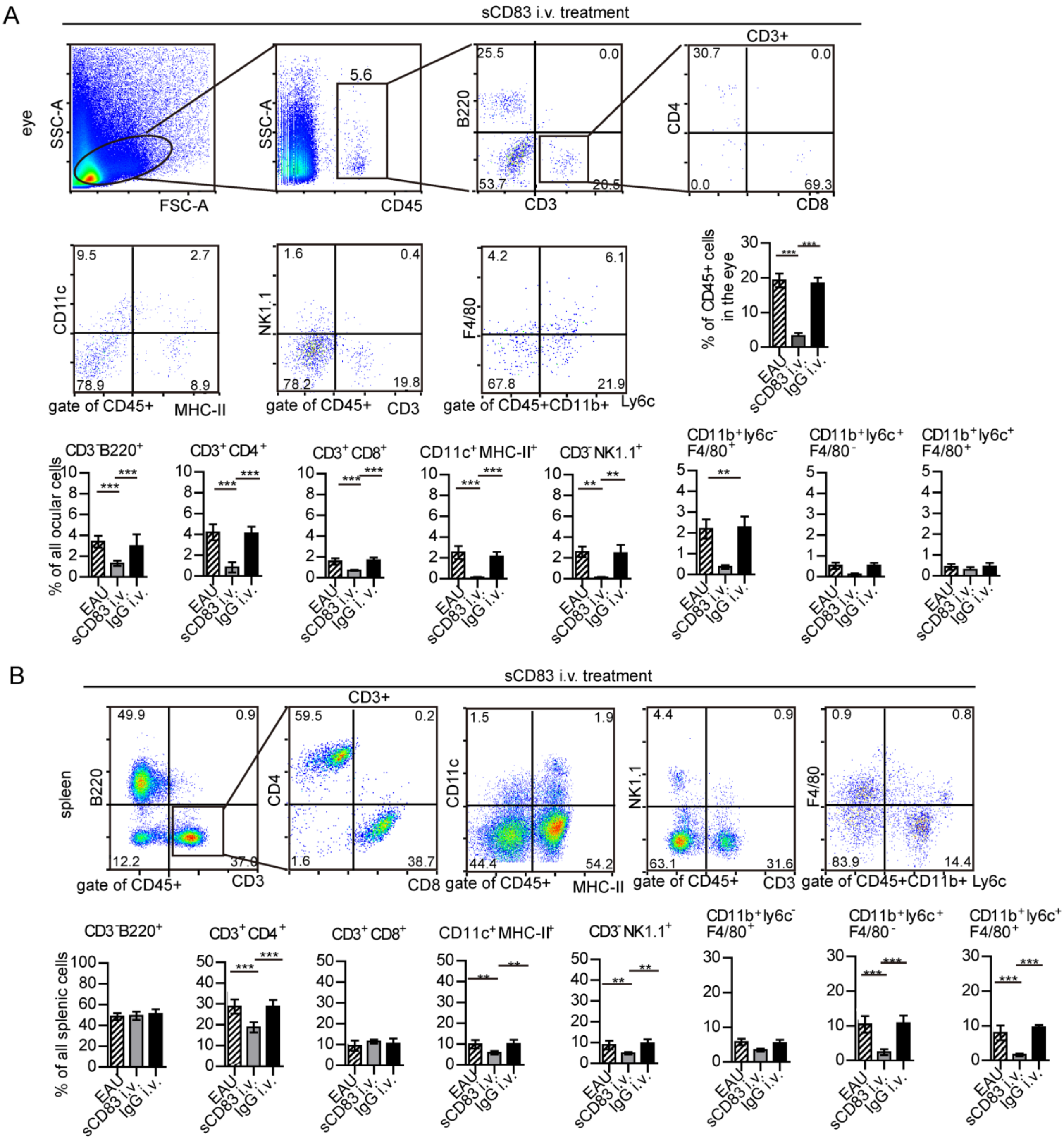

Supplemental Figure 10: The leukocyte landscape in the eye and spleen of EAU mice with systemic sCD83 (i.v.) treatment. Representative flow cytometry graphs and aggregated data of leukocyte subsets in the eye **(A)** and spleen **(B)** from sCD83- or IgG_1-treated (i.v.) EAU mice are shown. Mean ± s.e.m., n = 15 in triplicates, one-way ANOVA LSD-t test. **p*<0.05, ** *p*<0.01, and *** *p*<0.001.

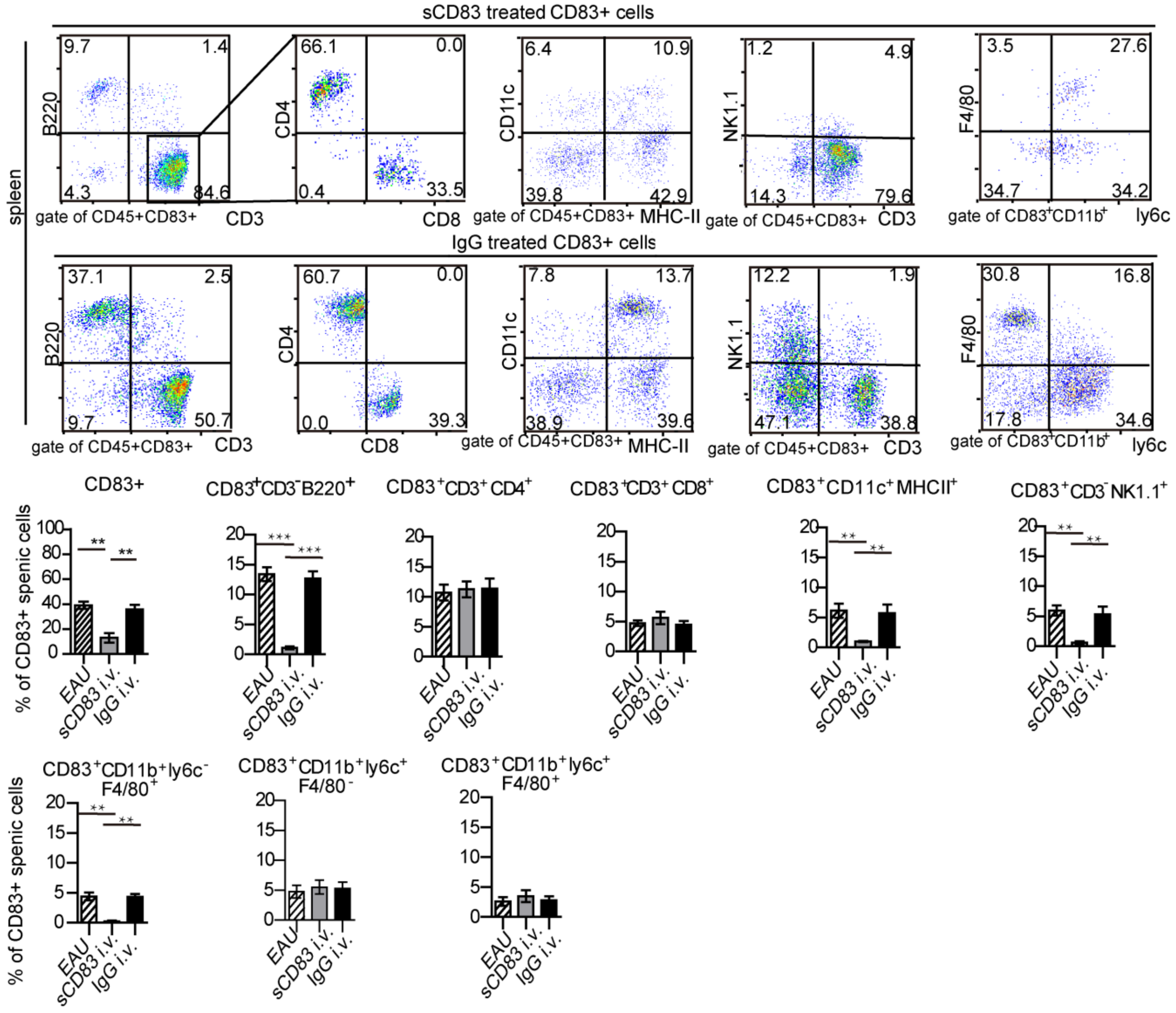

Supplemental Figure 11: Systemic sCD83 treatment reduces splenic $CD83^+$ leukocytes in EAU mice. After systemic sCD83 treatment (i.v.), the $CD83^+$ leukocyte subsets in the inflamed spleen were analyzed by flow cytometry. Untreated and IgG_1-treated mice served as controls. All data are shown as mean ± s.e.m., n=15 in triplicates, one-way ANOVA LSD-t test. **p*<0.05, ** *p*<0.01, and *** *p*<0.001.

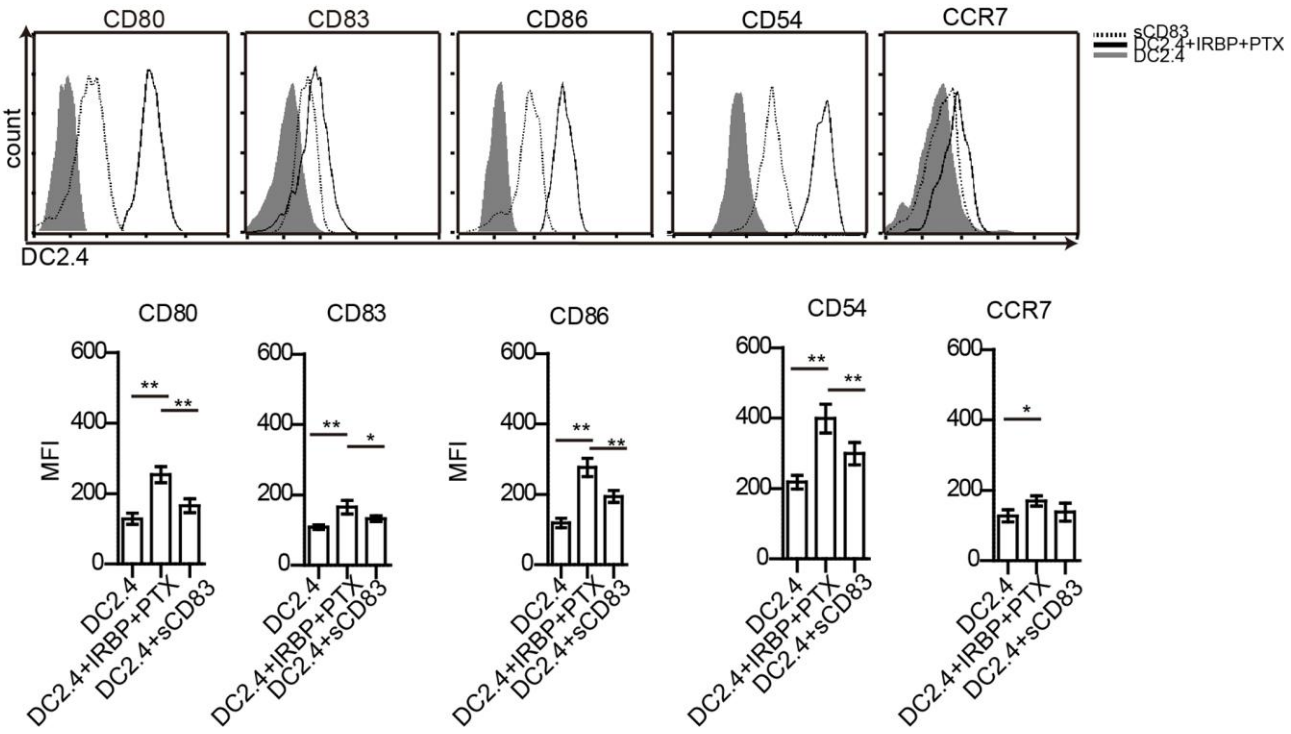

Supplemental Figure 12: The maturation of DC2.4 cells is impaired by sCD83 treatment. The surface expression of CD80, CD83, CD86, CD54, and CCR7 in DC2.4 cells, DC2.4 cells pulsed with $IRBP_{1\text{-}20}$ and PTX (to induce maturation) with and without sCD83 treatment was measured by flow cytometry (upper panel). The mean fluorescence intensity (MFI) is shown in the bottom panel as mean ± s.e.m. from three separate experiments. One-way ANOVA LSD-t test. *p<0.05, and ** p<0.01.

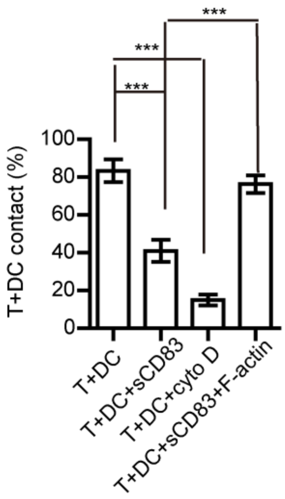

Supplemental Figure 13: The proportion of T-DCs contact. The proportion of T-DCs contact was measured by confocal microscopy. Three different perspectives were measured. The number of DC in contact with T cells/ all of DCs = T-DC contact (%). Untreated controls were compared to sCD83-, cytochalasin D-treated DC2.4 cells, and DC2.4 cells with F-actin overexpression. Mean ± s.e.m., three independent experiments were measured. *** $p<0.001$, One-way ANOVA LSD-t test.

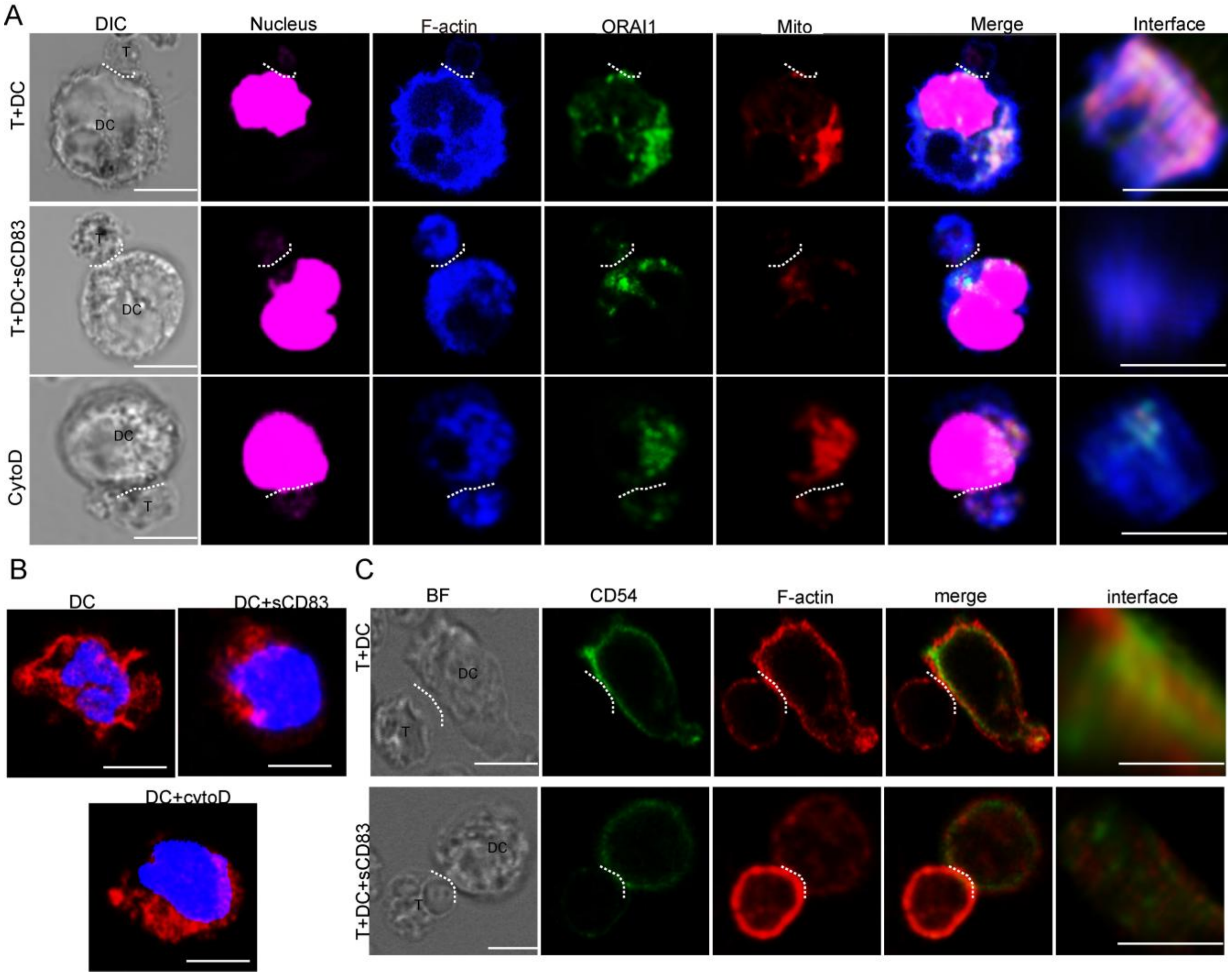

Supplemental Figure 14. sCD83 disrupts cytoskeletal F-actin and the topology of calcium microdomains in antigen-presenting wild type DCs. T-DC doublets were chosen from bright field (BF) images and evaluated using fluorescence image stacks. **(A)** Localization of F-actin (blue), ORAI1 (green) and mitochondria (red) at the contact zone of sCD83-treated wild type DC-T cells, untreated DC-T cells and cytochalasin D-treated DC-T cells. Scale bar = 5 μm. **(B)** The distribution of F-actin (red) in DCs, sCD83-pretreated DCs, cytochalasin D-pretreated DCs and sCD83-pretreated DC2.4 with F-actin overexpression. Scale bar = 5μm. The right panels show the enlarged view of the contact zone. A 3-dimensional image of DC-T contact was reconstructed. Scale bar = 2 μm. **(C)** Co-localization analysis of F-actin (red) and CD54 (green) at the wild type DC-T cell synapsis, and sCD83-treated DC-T synapsis. Scale bar = 5 μm. The dotted lines mark the DC-T synapse.

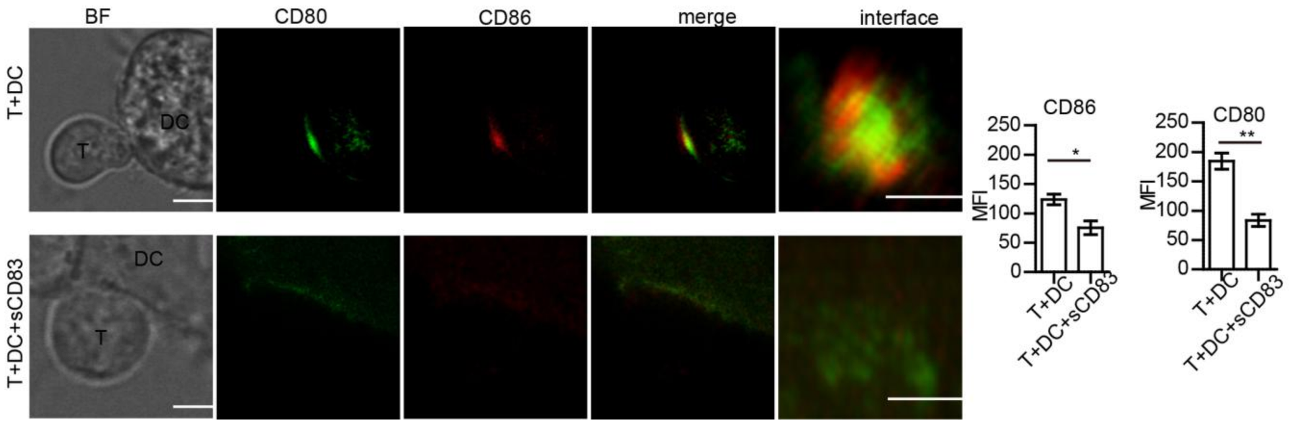

Supplemental Figure 15: sCD83 affects the localization of CD80, CD86 at the T-DC cell contact. The localization of CD80 (green) and CD86 (red) at the T-DC cell contact zone was analyzed by confocal microscopy with untreated (top row) or sCD83-treated DC2.4 (bottom row). Mean ±s.e.m. Scale bar=5 μm (mean ± s.e.m., n=25, from three separate experiments; * p <0.05 and ** p <0.01, two-tailed Student's t test).

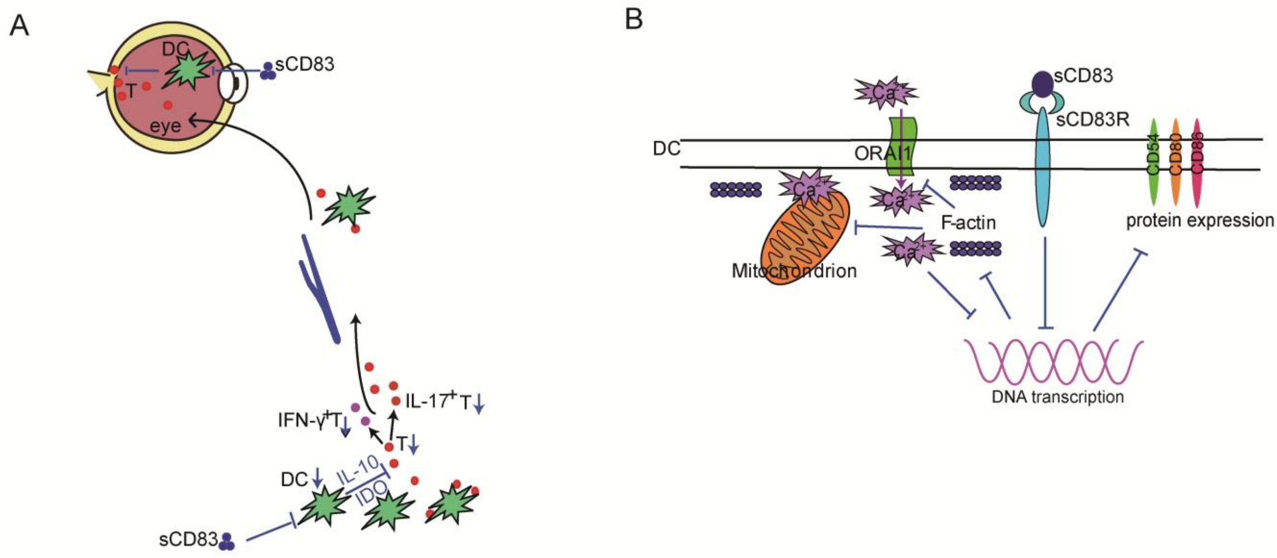

Supplemental Figure 16: A diagram of the effect of sCD83 treatment on EAU. (A) The possible effect of sCD83 on EAU. Intravenous sCD83 treatment or topical sCD83 treatment ameliorates EAU by inducing tolerogenic DCs to further inhibit T cells activation. **(B)** sCD83 binds its receptor (for example TLR4/MD-2)(3) to alter F-actin function(4) to affect calcium signaling and co-stimulatory molecules in DCs, leading to impair T cell activation.

REFERENCE

1. Harimoto K, Ito M, Karasawa Y, Sakurai Y, Takeuchi M. Evaluation of mouse experimental autoimmune uveoretinitis by spectral domain optical coherence tomography. *Br J Ophthalmol* (2014) 98: 808-812. doi: 10.1136/bjophthalmol-2013-304421.

2. Thurau SR, Chan CC, Nussenblatt RB, Caspi RR. Oral tolerance in a murine model of relapsing experimental autoimmune uveoretinitis (EAU): induction of protective tolerance in primed animals. *Clin Exp Immunol* (1997) 109: 370-376. doi: 10.1046/j.1365-2249.1997.4571356.x.

3. Horvatinovich, J. M., E. W. Grogan, M. Norris, A. Steinkasserer, H. Lemos, A. L. Mellor, I. Y. Tcherepanova, C. A. Nicolette, and M. A. DeBenedette. Soluble CD83 Inhibits T Cell Activation by Binding to the TLR4/MD-2 Complex on CD14+ Monocytes. *J Immunol* (2017). 198:2286-2301.

4. Kotzor, N., M. Lechmann, E. Zinser, and A. Steinkasserer. The soluble form of CD83 dramatically changes the cytoskeleton of dendritic cells. *Immunobiology* (2004),209:129-140.
